# The relationship between hippocampal-dependent task performance and hippocampal grey matter myelination and iron content

**DOI:** 10.1177/23982128211011923

**Published:** 2021-04-26

**Authors:** Ian A. Clark, Martina F. Callaghan, Nikolaus Weiskopf, Eleanor A. Maguire

**Affiliations:** 1 Wellcome Centre for Human Neuroimaging, UCL Queen Square Institute of Neurology, University College London, London, UK; 2 Department of Neurophysics, Max Planck Institute for Human Cognitive and Brain Sciences, Leipzig, Germany; 3 Felix Bloch Institute for Solid State Physics, Faculty of Physics and Earth Sciences, Leipzig University, Leipzig, Germany

**Keywords:** Hippocampus, quantitative MRI, scene construction, autobiographical memory, future thinking, spatial navigation, myelin, iron, tissue microstructure

## Abstract

Individual differences in scene imagination, autobiographical memory recall, future thinking and spatial navigation have long been linked with hippocampal structure in healthy people, although evidence for such relationships is, in fact, mixed. Extant studies have predominantly concentrated on hippocampal volume. However, it is now possible to use quantitative neuroimaging techniques to model different properties of tissue microstructure in vivo such as myelination and iron. Previous work has linked such measures with cognitive task performance, particularly in older adults. Here we investigated whether performance on scene imagination, autobiographical memory, future thinking and spatial navigation tasks was associated with hippocampal grey matter myelination or iron content in young, healthy adult participants. Magnetic resonance imaging data were collected using a multi-parameter mapping protocol (0.8 mm isotropic voxels) from a large sample of 217 people with widely-varying cognitive task scores. We found little evidence that hippocampal grey matter myelination or iron content were related to task performance. This was the case using different analysis methods (voxel-based quantification, partial correlations), when whole brain, hippocampal regions of interest, and posterior:anterior hippocampal ratios were examined, and across different participant sub-groups (divided by gender and task performance). Variations in hippocampal grey matter myelin and iron levels may not, therefore, help to explain individual differences in performance on hippocampal-dependent tasks, at least in young, healthy individuals.

## Introduction

Variations in hippocampal structure within the healthy population have long been posited to influence performance on tasks known to be hippocampal-dependent, such as scene imagination, autobiographical memory recall, future thinking and spatial navigation. Extant studies have predominantly examined this relationship in terms of hippocampal grey matter volume. However, in reviewing the literature, Clark et al. (2020) found mixed evidence for an association between hippocampal grey matter volume and performance on tasks assessing these cognitive functions in healthy individuals. They then proceeded to examine this issue in-depth by collecting data from a large sample of 217 young, healthy, adult participants but found little evidence that hippocampal grey matter volume was related to task performance (Clark et al., 2020).

It could be argued, however, that hippocampal grey matter volume is too blunt an instrument to consistently detect structure–function relationships in healthy young people. By contrast, it is now possible to use quantitative neuroimaging techniques to model different properties of tissue microstructure, such as myelination and iron content (Weiskopf et al., 2015). Myelin in brain tissue is thought to facilitate connectivity between neurons, with greater levels of myelination increasing the speed with which neurons can communicate (Nave and Werner, 2014). Iron is also important to consider as it is involved in the production and maintenance of myelin, and is therefore required for normal brain function (Mills et al., 2010; Todorich et al., 2009).

Myelination and iron content in grey matter can be studied in vivo in humans using a multi-parameter mapping (MPM) quantitative neuroimaging protocol (Callaghan et al., 2015, 2019; Weiskopf et al., 2013). Processing of these images using the hMRI toolbox (Tabelow et al., 2019) results in four maps that are differentially (but not solely) sensitive to specific aspects of tissue microstructure. These are magnetisation transfer saturation (MT saturation), sensitive to myelination; proton density (PD), sensitive to tissue water content; the longitudinal relaxation rate (R_1_), sensitive to myelination, iron and water content (but primarily myelination); and the effective transverse relaxation rate (R_2_^*^), sensitive to tissue iron content. Extant studies have reported relationships between myelination, iron and ageing across the lifespan (Callaghan et al., 2014; Draganski et al., 2011) and in young adults (Carey et al., 2018), and also correlations with verbal memory performance in older adults (Steiger et al., 2016), and meta-cognitive ability in young adults (Allen et al., 2017). However, as far as we are aware, no studies have investigated the relationship between hippocampal grey matter myelination or iron content and scene imagination, autobiographical memory recall, future thinking or navigation ability in healthy young people. Consequently, this is what we sought to examine in this study.

We used the large data set (n = 217) from Clark et al.’s (2020) study that comprised an MPM quantitative imaging protocol (Callaghan et al., 2015, 2019; Weiskopf et al., 2013), and cognitive task performance with wide variability in scores. While aspects of these data (hippocampal grey matter volume, cognitive task performance) have been reported before (Clark et al., 2019, 2020; Clark and Maguire, 2020), the tissue microstructure MRI data, measuring myelination and iron content, have not been published previously. The mixed literature relating to hippocampal grey matter volume and the dearth of studies investigating hippocampal grey matter myelination and iron content made the formulation of clear hypotheses difficult. As such, we focussed on conducting deep and wide-ranging data analyses to characterise any links between the tissue microstructure measures and task performance in the same manner as Clark et al. (2020).

## Methods

### Participants

Two hundred and seventeen people (mean age 29.0 years ± 5.60) were recruited from the general population, 109 females and 108 males. The age range was restricted to 20–41 years to limit the possible effects of ageing. Participants had English as their first language and reported no psychological, psychiatric or neurological health conditions. People with hobbies or vocations known to be associated with the hippocampus (e.g. licenced London taxi drivers) were excluded. Participants were reimbursed £10 per hour for taking part that was paid at study completion. All participants gave written informed consent and the study was approved by the University College London Research Ethics Committee (project ID: 6743/001).

### Procedure

Participants completed the study over three visits – structural MRI scans were acquired during the first visit, and cognitive testing was conducted during visits 2 and 3. All participants completed all parts of the study.

### Cognitive tasks and statistical analyses

All tasks are published and were performed and scored as per their published use. Full descriptions are also provided in Clark et al. (2019, 2020) and Clark and Maguire (2020). Details of the double scoring for this study are provided in the Supplemental Material Tables S1–S4. In brief, there were four tasks: (1) Scene imagination was tested using the scene construction task (Hassabis et al., 2007) which measures the ability to mentally construct visual scenes. The main outcome measure is the ‘experiential index’, while the sub-measures of interest are content scores and a rating of the spatial coherence of scenes. (2) Autobiographical memory recall was tested using the autobiographical interview (AI; Levine et al., 2002), which measures the ability to recall past experiences over four time periods from early childhood to within the last year. The two main outcome measures are the number of ‘internal’ and ‘external’ details. Internal details are of interest here because they describe the event in question (i.e. episodic details) and are thought to be hippocampal dependent. Sub-measures are the separate content categories that comprise the internal details outcome measure, and also AI vividness ratings. (3) The future thinking task follows the same procedure as the scene construction task, but requires participants to imagine three plausible future scenes involving themselves (an event at the weekend; next Christmas; the next time they will meet a friend). (4) Navigation ability was assessed using the paradigm described by Woollett and Maguire (2010). A participant watches movie clips of two overlapping routes through an unfamiliar real town four times. The main outcome measure is the combined scores from the five sub-measures used to assess navigational ability which are: movie clip recognition, recognition memory for scenes, landmark proximity judgements, route knowledge where participants place scene photographs from the routes in the correct order as if travelling through the town, and the drawing of a sketch map. Data were summarised using means and standard deviations, calculated in SPSS, version 22.

### MRI data acquisition and preprocessing

Three Siemens Magnetom TIM Trio MRI systems with 32 channel head coils were used to collect the structural neuroimaging data. All scanners were located at the same imaging centre, running the same software.

Whole brain images at an isotropic resolution of 800 μm were obtained using an MPM quantitative imaging protocol (Callaghan et al., 2015, 2019; Weiskopf et al., 2013). This consisted of the acquisition of three multi-echo gradient acquisitions with either PD, T1 or MT weighting. Each acquisition had a repetition time, TR, of 25 ms. PD weighting was achieved with an excitation flip angle of 6°, that was increased to 21° to achieve T1 weighting. MT weighting was achieved through the application of a Gaussian RF pulse 2 kHz off resonance with 4 ms duration and a nominal flip angle of 220°. This acquisition had an excitation flip angle of 6°. The field of view was 256 mm head-foot, 224 mm anterior-posterior (AP), and 179 mm right-left (RL). The multiple gradient echoes per contrast were acquired with alternating readout gradient polarity at eight equidistant echo times ranging from 2.34 to 18.44 ms in steps of 2.30 ms using a readout bandwidth of 488 Hz/pixel. Only six echoes were acquired for the MT-weighted volume to facilitate the off-resonance pre-saturation pulse within the TR. To accelerate the data acquisition, partially parallel imaging using the GRAPPA algorithm was employed in each phase-encoded direction (AP and RL) with 40 integrated reference lines and a speed up factor of two. Calibration data were also acquired at the outset of each session to correct for inhomogeneities in the RF transmit field (Lutti et al., 2010, 2012).

**Figure 1.**
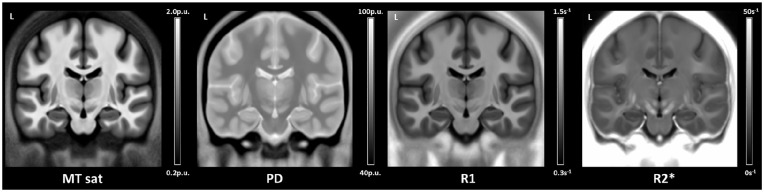
The averaged magnetisation transfer saturation (MT sat), proton density (PD), longitudinal relaxation rate (R_1_) and effective transverse relaxation rate (R_2_^*^) tissue microstructure maps of the whole sample (n = 217) in MNI space. The scale bars are the estimated physical values of the tissue properties in each map quantified in standardised units. For the MT saturation and PD maps, this is as percent units (p.u.), and for the R_1_ and R_2_* maps, this is per second (s^–1^).

Data were processed using the hMRI toolbox (Tabelow et al., 2019) within SPM12 (www.fil.ion.ucl.ac.uk/spm). The default toolbox configuration settings were used, with the exception that correction for imperfect spoiling was additionally enabled (see also Callaghan et al., 2019). As alluded to earlier, this image processing resulted in four maps that differentially modelled tissue microstructure (Figure 1): an MT saturation map sensitive to myelination, a PD map sensitive to tissue water content, an R_1_ map sensitive to myelination, iron and water content (but primarily myelination), and an R_2_^*^ map sensitive to tissue iron content.

Each participant’s MT saturation map was then segmented into grey and white matter probability maps using the unified segmentation approach (Ashburner and Friston, 2005), but using the tissue probability maps developed by Lorio et al. (2016) and no bias field correction (since the MT saturation map shows negligible bias field modulation). The grey and white matter probability maps were used to perform inter-subject registration using DARTEL, a nonlinear diffeomorphic algorithm (Ashburner, 2007). The resulting DARTEL template and deformations were used to normalise the MT saturation, PD, R_1_ and R_2_* maps to the stereotactic space defined by the Montreal Neurological Institute (MNI) template (at 1 × 1 × 1 mm resolution), but without modulating by Jacobian determinants of the deformation field in order to allow for the preservation of the quantitative values. Finally, a tissue-weighted smoothing kernel of 4 mm full width at half maximum (FWHM) was applied using the voxel-based quantification (VBQ) approach (Draganski et al., 2011), which again aims to preserve the quantitative values.

### Assessing basic tissue properties

We first examined basic properties of the tissue microstructure maps to assess whether they were in line with expectations. In healthy individuals, myelination levels have been reported to be higher in primary sensory regions (Carey et al., 2018; Dick et al., 2012), while iron levels are increased in the substantia nigra and red nucleus (Beard et al., 1993; Drayer et al., 1986). To test that this was also the case for the current data, regions of interest (ROIs) of Heschl’s gyrus (containing the primary auditory cortex), the substantia nigra and the red nucleus were defined using the Automated Anatomical Labelling Atlas v3 (Rolls et al., 2020). The Heschl’s gyrus mask was applied to each participant’s smoothed and normalised grey matter MT saturation map, and the substantia nigra and the red nucleus masks were applied to the smoothed and normalised grey matter R_2_* map. Average values within each mask were extracted using ‘spm_summarise’. Mean MT and R_2_* values were also extracted from across the whole of the grey matter using the grey matter mask defined for the VBQ analyses.

Two-tailed paired t-tests, implemented in SPSS v25 and thresholded at p < 0.001, were then performed between the values from the ROIs and the mean of the grey matter, with higher MT values predicted for the Heschl’s gyrus mask and higher R_2_* values for the substantia nigra and red nucleus masks when compared to the mean of the grey matter. Effect sizes are reported as Hedge’s g_av_, which is Cohen’s d calculated for repeated measures and corrected for the positive bias caused by using sample estimates (Lakens, 2013).

We found that MT saturation values were significantly higher in Heschl’s gyrus (mean MT = 0.90, SD = 0.035) compared to the mean of the grey matter (mean MT = 0.88, SD = 0.031; t_(216)_ = 15.90, p < 0.001; Hedge’s g_av_ = 0.64). In addition, R_2_* values were significantly higher in the substantia nigra (mean R_2_* = 26.76, SD = 3.21; t_(216)_ = 48.03, p < 0.001; Hedge’s g_av_ = 4.20) and red nucleus (mean R_2_* = 23.95, SD = 3.05; t_(216)_ = 35.28, p < 0.001; Hedge’s g_av_ = 3.14) compared to the mean of the grey matter (mean R_2_* = 17.03, SD = 0.63). Overall, therefore, higher levels of MT and R_2_* were observed in regions known to have high levels of myelination and iron, respectively, showing that our tissue microstructure maps aligned with expectations.

### Relationships between the tissue microstructure maps

Of note, the tissue microstructure maps are not completely independent since they are estimated from the same three multi-echo gradient echo acquisitions. As such, relationships exist between the tissue microstructure maps, and a finding in one map can be used to validate a finding in another. For example, if a positive association is observed between task performance and the hippocampus in the MT saturation map, then a corresponding positive association would also be expected in the hippocampus when using the R_1_ map (since increased macromolecular content will also increase R_1_), and a corresponding negative association would be expected in the PD map (due to a reduction in free water content as the macromolecular content increases; Mezer et al., 2013).

### Primary VBQ analyses

Our analyses followed the same procedures as those detailed by Clark et al. (2020), except that here we assessed each of the tissue microstructure maps using VBQ (Draganski et al., 2011). VBQ is a similar methodology to the voxel-based morphometry (VBM) technique used to study grey matter volume (Ashburner and Friston, 2000) but one that retains the quantitative values carrying information about the tissue microstructure.

First, we examined the relationship between hippocampal grey matter in each of the tissue microstructure maps and the main outcome measure for each of the cognitive tasks assessing scene imagination, autobiographical memory, future thinking and navigation. We then examined the associations between each of the sub-measures from these tasks and hippocampal grey matter in each of the four tissue microstructure maps. Statistical analyses were carried out using multiple linear regression models with cognitive task performance as the measure of interest, while including covariates for age, gender, total intracranial volume, and the different MRI scanners. The dependent variable was the smoothed and normalised grey matter value from each tissue microstructure map.

Whole brain VBQ analyses were carried out for each tissue microstructure map using an explicitly defined mask that was generated by averaging the smoothed and normalised MT saturation grey matter probability map across all participants. When the grey matter probability was below 80%, these voxels were excluded from the analysis. Two-tailed t-tests were used, with statistical thresholds applied at p < 0.05 family-wise error (FWE) corrected for the whole brain, and a minimum cluster size of 5 voxels.

As relationships exist between the tissue microstructure maps, following the finding of a relationship in one map, we also examined whether corresponding relationships existed in the other maps, even at a more liberal statistical threshold (p < 0.001 uncorrected). Observing related associations across multiple maps was deemed supportive of a true result, while finding a correlation in only one of the tissue microstructure maps was regarded as unreliable.

### Hippocampal ROI VBQ

Following the whole brain analysis, we focused on the hippocampus using bilateral anatomical hippocampal masks. The masks were manually delineated on the group-averaged MT saturation map in MNI space (1 × 1 × 1 mm) using ITK-SNAP (www.itksnap.org). As in Poppenk and Moscovitch (2011) and Brunec et al. (2019), the anterior hippocampus was delineated using an anatomical mask that was defined in the coronal plane and proceeded from the first slice where the hippocampus can be observed in its most anterior extent until the final slice of the uncus. The posterior hippocampus was defined from the first slice following the uncus until the final slice of observation in its most posterior extent. The whole hippocampal mask comprised the combination of the anterior and posterior masks. ROI analyses were performed using two-tailed t-tests, with voxels regarded as significant when falling below an initial whole brain uncorrected voxel threshold of p < 0.001, and then a small volume correction threshold of p < 0.05 FWE corrected for each mask, with a minimum cluster size of 5 voxels. As with the whole brain analyses, following the finding of a relationship in one map, we also examined whether corresponding relationships existed in the other maps, even at a more liberal statistical threshold of p < 0.001 uncorrected.

### Auxiliary analyses using extracted hippocampal microstructure measurements

Several auxiliary analyses were performed using the hippocampal grey matter tissue microstructure measurements that were extracted for each participant from each tissue microstructure map using ‘spm_summarise’. Whole, anterior and posterior bilateral anatomical hippocampal masks were applied to each participant’s smoothed and normalised grey matter MT saturation, PD, R_1_, and R_2_* maps, and the average value within each mask extracted. We also calculated each participant’s posterior: anterior hippocampal ratio for each tissue microstructure measurement (Poppenk and Moscovitch, 2011).

We first performed partial correlations between the extracted tissue microstructure metrics and the cognitive task performance measures. Then, we investigated the effects of gender. Next, we examined the effects of task performance by dividing the sample into low- and high-performing groups dependent on the median score for each task, directly comparing the two groups. Finally, we also compared the best and worst performers for each task, defined as approximately the top and bottom 10% (n ~ 20 in each group). Age, gender, total intracranial volume and MRI scanner were included as covariates in all analyses, with the exception of those examining gender, where gender was not a covariate. As in Clark et al. (2020), statistical correction was made using false discovery rate (FDR; Benjamini and Hochberg, 1995), with an FDR of p < 0.05 allowing for 5% false-positive results across the tests performed and calculated using the resources provided by McDonald (2014).

## Results

### Cognitive task performance

A summary of the outcome measures for the cognitive tasks is shown in Table 1. A wide range of scores was obtained for all variables with the exception of navigation movie clip recognition, where performance was close to ceiling.

**Table 1.** Means and standard deviations for the main outcome measures and sub-measures of the cognitive tasks.

Variable	Mean	Standard deviation
Main outcome measures		
Scene construction experiential index (/60)	40.50	6.08
Autobiographical interview internal details (total number)	23.95	7.25
Future thinking experiential index (/60)	39.12	7.23
Navigation (/250)	143.46	35.83
Sub-measures		
Scene construction		
Spatial references (total number)	3.47	1.56
Entities present (total number)	9.90	2.92
Sensory descriptions (total number)	12.25	3.46
Thoughts/emotions/actions (total number)	3.36	1.70
Spatial coherence index (/6)	2.88	1.57
Autobiographical interview		
Internal events (total number)	11.02	3.88
Internal time (total number)	1.43	0.69
Internal place (total number)	2.23	0.63
Internal perceptual (total number)	5.91	3.07
Internal thoughts/emotions (total number)	3.37	1.69
Vividness ratings (/6)	4.62	0.74
Future thinking		
Spatial references (total number)	2.53	1.65
Entities present (total number)	10.31	3.28
Sensory descriptions (total number)	8.81	3.59
Thoughts/emotions/actions (total number)	5.18	2.28
Spatial coherence index (/6)	2.63	1.77
Navigation		
Movie clip recognition (/16)	15.44	0.86
Scene recognition (/32)	29.45	1.89
Proximity judgements (/10)	7.48	1.43
Route knowledge (/24)	11.98	4.62
Sketch map	79.11	30.91

### Primary VBQ analyses

As our main focus was on the relationship between cognitive task performance and hippocampal grey matter myelination and iron content, here we report findings pertaining to the hippocampus – any regions identified in the whole brain analysis that were outside the hippocampus are reported in the Supplemental Results.

No significant relationships were evident between cognitive task performance and hippocampal grey matter in any of the tissue microstructure maps. This was the case for the main outcome measures of the tasks assessing scene imagination, autobiographical memory, future thinking or navigation, and also for the sub-measures of these tasks.

### Hippocampal ROI VBQ

On examination of the hippocampal masks, no relationships were evident between cognitive task performance and hippocampal grey matter in any of the tissue microstructure maps. Considering the task sub-measures, it was either the case that they were not associated with any measure of hippocampal grey matter tissue microstructure, or the results were not validated across the tissue microstructure maps – correlations associated with only one map are reported in the Supplemental Results.

### Auxiliary analyses using extracted hippocampal microstructure measurements

The means and standard deviations of the extracted hippocampal grey matter tissue microstructure metrics are reported in the Supplemental Results, Table S5.

No relationships were identified between any of the extracted hippocampal grey matter tissue microstructure metrics and performance on any of the main (Table 2) or sub-measures of the cognitive tasks (Supplemental Results Tables S6–S9). These partial correlation findings therefore support those of the primary VBQ analyses.

**Table 2.** Partial correlations between the main outcome measures of the scene construction, autobiographical memory, future thinking and navigation tasks and the extracted hippocampal grey matter tissue microstructure metrics, with age, gender, total intracranial volume and MRI scanner as covariates.

Tissue microstructure maps and task measures	Whole hippocampus		Anterior hippocampus		Posterior hippocampus		Posterior/anterior hippocampus ratio	
	r	p	r	p	r	p	r	p
MT saturation								
Scene construction EI	−0.096	0.59	−0.097	0.59	−0.081	0.59	0.043	0.74
AI internal details	−0.005	0.99	0.002	0.99	−0.012	0.99	−0.026	0.99
Future thinking EI	−0.14	0.31	−0.13	0.31	−0.13	0.31	0.05	0.81
Overall navigation score	−0.11	0.91	−0.071	0.96	−0.14	0.91	−0.058	0.96
PD								
Scene construction EI	−0.028	0.83	−0.044	0.73	−0.001	0.99	0.053	0.72
AI internal details	0.045	0.99	0.093	0.85	−0.026	0.99	−0.14	0.75
Future thinking EI	−0.039	0.87	−0.031	0.90	−0.038	0.87	0.005	0.98
Overall navigation score	−0.012	0.99	−0.018	0.99	−0.001	0.99	0.022	0.99
R_1_								
Scene construction EI	0.092	0.59	0.11	0.59	0.065	0.67	−0.082	0.59
AI internal details	0.13	0.77	0.091	0.85	0.15	0.75	0.08	0.88
Future thinking EI	0.13	0.31	0.12	0.34	0.13	0.31	0.006	0.98
Overall navigation score	−0.014	0.99	0.002	0.99	−0.026	0.99	−0.045	0.99
R_2_*								
Scene construction EI	0.082	0.59	0.059	0.67	0.086	0.59	0.004	0.98
AI internal details	0.029	0.99	0.002	0.99	0.049	0.99	0.034	0.99
Future thinking EI	0.12	0.35	0.11	0.37	0.099	0.40	−0.032	0.90
Overall navigation score	0.065	0.96	0.018	0.99	0.097	0.91	0.095	0.91

MT saturation: magnetisation transfer saturation; EI: Experiential Index; AI: Autobiographical Interview; PD: proton density; R_1_: longitudinal relaxation rate; R_2_*: effective transverse relaxation rate.

P values are Benjamini–Hochberg false discovery rate corrected at p < 0.05.

Similarly, there were no significant effects of gender (Supplemental Results Tables S10–S17), and no significant findings from the median split direct comparisons (Supplemental Results Tables 18–22), partial correlations (Supplemental Results Tables S23–S30), and when the best and worst performers were compared (Supplemental Results Tables 31–35).

## Discussion

In this study, we moved beyond hippocampal grey matter volume to examine hippocampal grey matter tissue microstructure in the form of quantitative neuroimaging biomarkers of myelination and iron content, and whether they were linked with performance on tasks known to be hippocampal-dependent. We found little evidence for any associations between these measures and scene imagination, autobiographical memory recall, future thinking and spatial navigation. This was despite having a large sample with wide-ranging scores on the cognitive tasks, using different analysis methods (VBQ and partial correlations), examining whole brain and hippocampal ROIs (bilateral whole hippocampus, anterior and posterior portions and hippocampal posterior:anterior ratio) and different participant sub-groups (divided by gender and task performance). Variations in hippocampal grey matter myelination or iron content, seem not, therefore, to be significantly related to hippocampal-dependent task performance in young, healthy individuals.

Myelination and iron are essential for communication between neurons (Nave and Werner, 2014; Todorich et al., 2009). Therefore, it could have been that higher levels of myelin within the hippocampus would result in faster hippocampal neuronal communication, enabling better task performance. Conversely, it might have been the case that higher levels of hippocampal iron would be associated with lower task performance – while moderate iron levels are required for normal functioning, excessive iron accumulation can impede function (Zecca et al., 2004). Indeed, such a finding has previously been reported in young healthy adults in relation to meta-cognitive ability (Allen et al., 2017). Here, however, we found no relationships between hippocampal grey matter myelination or iron content and performance on scene imagination, autobiographical memory recall, future thinking or spatial navigation tasks, aligning with previous null results relating to hippocampal grey matter volume more generally (Clark et al., 2020; Maguire et al., 2003; Van Petten, 2004; Weisberg et al., 2019).

Non-significant results can, of course, be difficult to interpret, and an absence of evidence is not necessarily evidence of absence. However, we believe the depth and breadth of our analyses permit confidence in these results. First, we had a large sample size with wide variance on our measures of interest, both in terms of task performance (with the exception of the navigation movie clip recognition sub-measure where performance was close to ceiling), and the tissue microstructure metrics. We were, therefore, well-placed to identify potential relationships between hippocampal grey matter myelination and iron content and cognitive task performance. Second, we used two different analysis methodologies, as well as employing bilateral hippocampal ROIs, and even divided the sample into multiple sub-groups. Regardless of these different approaches to data analysis, no reliable associations between hippocampal grey matter myelination or iron content and task performance were identified. The results, therefore, do not seem to be consequent upon a specific analysis technique or sub-group of participants, but instead there was a consistent pattern of non-significant results. Moreover, we also ascertained that the tissue microstructure maps were in line with expectations. As predicted, higher levels of myelination were identified in Heschl’s gyrus (which contains the primary auditory cortex), and greater levels of iron were observed in the substantia nigra and red nucleus. The non-significant results cannot, therefore, be explained by basic issues with the neuroimaging data.

That is not to say that relationships between hippocampal grey matter myelination or iron content and performance on our cognitive tasks of interest may not exist in other contexts. In older participants, for example, associations between hippocampal grey matter volume and performance on autobiographical and navigation tasks have been identified (e.g. Hedden et al., 2014; Moffat et al., 2006), despite less reliable evidence for such relationships in healthy young adults (Clark et al., 2020; Maguire et al., 2003; Van Petten, 2004; Weisberg et al., 2019). Changes in the extent of grey matter myelination and iron content have been documented with ageing (e.g. Callaghan et al., 2014; Carey et al., 2018; Draganski et al., 2011), as has a link between poorer verbal memory performance, decreased myelin content and increased levels of iron in the ventral striatum in older adults (Steiger et al., 2016). Therefore, similar investigations to those performed here using an older sample could reveal associations not observed in this study.

Similarly, relationships between hippocampal grey matter myelination or iron content and task performance might only be apparent in special populations. The extreme spatial navigation of licenced London taxi drivers who have acquired ‘The Knowledge’ of London’s layout has been reliably associated with increased posterior, but decreased anterior, hippocampal grey matter volume (e.g. Maguire et al., 2000, 2006; Woollett et al., 2008; Woollett and Maguire, 2011). Variations may also exist, therefore, in hippocampal grey matter myelination and iron content, and these differences may help to elucidate how a larger posterior hippocampus might be beneficial to London taxi drivers. Comparisons between healthy individuals and patients with memory loss may also reveal important differences in the extent of myelination and iron content in the hippocampus but also elsewhere in the brain.

The hippocampus is composed of distinct subfields, and myelination or iron content in specific subregions may be associated with task performance, as opposed to associations being apparent at the level of the whole hippocampus. Some evidence exists to suggest this may be the case for hippocampal grey matter volume (Barry et al., 2021; Chadwick et al., 2014; Palombo et al., 2018), and future studies could investigate relationships between task performance and subfield-specific hippocampal grey matter tissue microstructure.

It is also possible to obtain different measures of tissue microstructure to those studied here. Using diffusion MRI, the fractional anisotropy (FA) of right hippocampal grey matter has been associated with navigation ability (Iaria et al., 2008), and the FA and mean diffusivity of various white matter pathways, including the fornix, have been linked with autobiographical memory, future thinking and navigation performance (Hodgetts et al., 2017, 2020; Irish et al., 2014; Metzler-Baddeley et al., 2011; Williams et al., 2020). While replication of these results in larger samples is required, examining these tissue microstructure metrics, or white matter instead of grey matter, may reveal relationships between brain tissue microstructure and performance that were not evident in this study. Moreover, by combining diffusion MRI and the quantitative neuroimaging techniques used here additional, more biologically interpretable microstructure measurements, such as the axonal g-ratio (e.g. Mohammadi et al., 2015), can be obtained, opening up further avenues of investigation to understand the neural basis of individual differences in cognition.

In conclusion, having tested a large sample of young healthy participants with a wide range of scores on multiple cognitive tasks, no credible associations between hippocampal grey matter myelination or iron content and scene imagination, autobiographical memory, future thinking and spatial navigation performance were identified. Consequently, variability in hippocampal grey matter myelination or iron content seem unlikely to explain individual differences in ability, at least in the general population of young healthy people. Further investigation is required to establish whether such measures may be informative in other cohorts, as well as combining myelination and iron content with other tissue microstructure techniques to examine the basis of individual differences in key aspects of cognition including recalling the past and imagining the future.

## Supplemental Material

sj-docx-1-bna-10.1177_23982128211011923 – Supplemental material for The relationship between hippocampal-dependent task performance and hippocampal grey matter myelination and iron content Click here for additional data file. Supplemental material, sj-docx-1-bna-10.1177_23982128211011923 for The relationship between hippocampal-dependent task performance and hippocampal grey matter myelination and iron content by Ian A. Clark, Martina F. Callaghan, Nikolaus Weiskopf and Eleanor A. Maguire in Brain and Neuroscience Advances
