# Supplementary material for: The relationship between hippocampal-dependent task performance and hippocampal grey matter myelination and iron content
Source: Brain Neurosci Adv. 2021 Apr 26;5:23982128211011923. doi: 10.1177/23982128211011923 (PMC8079931; doi:10.1177/23982128211011923)
Supplement: sj-docx-1-bna-10.1177_23982128211011923 – Supplemental material for The relationship between hippocampal-dependent task performance and hippocampal grey matter myelination and iron content [file sj-docx-1-bna-10.1177_23982128211011923.docx]

**Supplemental Material for:**

**The relationship between hippocampal-dependent task performance and hippocampal grey matter myelination and iron content**

**Ian A. Clark^1^, Martina F. Callaghan^1^, Nikolaus Weiskopf^1,2,3^, and**

**Eleanor A. Maguire^1*^**

^1^Wellcome Centre for Human Neuroimaging, UCL Queen Square Institute of Neurology,

University College London, London, UK

^2^Department of Neurophysics, Max Planck Institute for Human Cognitive and Brain

Sciences, Leipzig, Germany

^3^Felix Bloch Institute for Solid State Physics, Faculty of Physics and Earth Sciences, Leipzig University, Leipzig, Germany

*Corresponding author:

Eleanor Maguire

[e.maguire@ucl.ac.uk](mailto:e.maguire@ucl.ac.uk)

**Supplemental Methods**

Double scoring was performed on 20% of the cognitive data. We took the most stringent approach to identifying across-experimenter agreement. Inter-class correlation coefficients with a two-way random effects model looked for absolute agreement among the experimenters. For reference, a score of 0.8 or above is considered excellent agreement beyond chance.

**Table S1.** Double scoring of the scene construction task.

|  | | Rating | | | | |
| --- | --- | --- | --- | --- | --- | --- |
|  | Spatial References | | Entities Present | Sensory Descriptions | Thoughts/  Emotions/Actions | Quality Ratings |
| **For each individual scene** | | | | | | |
|  |  | |  |  |  |  |
| n = 308 | 0.90 | | 0.96 | 0.94 | 0.90 | 0.90 |
|  |  | |  |  |  |  |
| **For each individual participant (i.e. score is averaged across the seven scenes)** | | | | | | |
|  | | | | | | |
| n = 44 | 0.91 | | 0.99 | 0.97 | 0.91 | 0.93 |
|  |  | |  |  |  |  |

Inter-class correlation coefficients from a two way random effect model looking for absolute agreement for each content score and for the quality ratings. Four experimenters scored the whole data set (n = 217 participants, 1519 individual scenes) with double scoring performed on 20% of the data (n = 44 participants, 308 scenes) proportionally for each original experimenter.

**Table S2.** Double scoring of the autobiographical interview.

|  | | Rating | | | | | | | | | | | |
| --- | --- | --- | --- | --- | --- | --- | --- | --- | --- | --- | --- | --- | --- |
|  | Internal Event | | | Internal Place | | Internal Time | | Internal Perceptual | | Internal Emotion | | Internal Sum |  |
| **For each individual memory** | | | | | | | | | | |  | |  |
|  |  | |  | |  | |  | |  | |  | |  |
| n = 215 | 0.92 | | | 0.85 | | 0.94 | | 0.92 | | 0.86 | | 0.94 |  |
|  |  | |  | |  | |  | |  | |  | |  |
| **For each individual participant (i.e. score is averaged across the five memories)** | | | | | | | | | | | | | |
|  | | | | | | | | | | |  | |  |
| n = 43 | 0.95 | | | 0.88 | | 0.96 | | 0.94 | | 0.81 | | 0.97 |  |
|  |  | |  | |  | |  | |  | |  | |  |

Inter-class correlation coefficients from a two way random effects model looking for absolute agreement for each score on the autobiographical interview. Three experimenters scored the whole data set (n = 217 participants, 1085 individual memories) and double scoring was performed 20% of the data (n = 43 participants, 215 individual memories) proportionally for each original experimenter.

**Table S3.** Double scoring of the future thinking task.

|  | | Rating | | | | |
| --- | --- | --- | --- | --- | --- | --- |
|  | Spatial References | | Entities Present | Sensory Descriptions | Thoughts/  Emotions/Actions | Quality Ratings |
| **For each individual scene** | | | | | | |
|  |  | |  |  |  |  |
| n = 132 | 0.90 | | 0.94 | 0.93 | 0.88 | 0.90 |
|  |  | |  |  |  |  |
| **For each individual participant (i.e. score is averaged across the three future scenes)** | | | | | | |
|  | | | | | | |
| n = 44 | 0.94 | | 0.95 | 0.96 | 0.88 | 0.92 |
|  |  | |  |  |  |  |

Inter-class correlation coefficients from a two way random effects model looking for absolute agreement for each content score and for the quality ratings. Four experimenters scored the whole data set (n = 217 participants, 651 individual future scenes) with double scoring performed on 20% of the data (n = 44 participants, 132 future scenes) proportionally for each original experimenter.

**Table S4.** Double scoring of the navigation sketch maps.

|  | Rating | | | | | | |
| --- | --- | --- | --- | --- | --- | --- | --- |
|  | | Road Segments | Road Junctions | Number of Landmarks | Landmark Placement | Map Orientation | Map Categorisation |
|  | |  |  |  |  |  |  |
| n = 42 | | 0.95 | 0.96 | 0.97 | 0.96 | 0.96 | 0.89 |
|  | |  |  |  |  |  |  |

Inter-class correlation coefficients from a two way random effects model looking for absolute agreement for each score on the navigation sketch maps. Three experimenters scored the whole data set (n = 217) and double scoring was performed on 20% of the data (n = 42 participants) proportionally for each original experimenter.

**Supplemental Results**

**Primary analyses: VBQ results outside of the hippocampus**

A small number of relationships were identified between cognitive task performance and the grey matter tissue microstructure maps outside of the hippocampus (when using a statistical threshold of p < 0.05 FWE whole brain corrected).

Within the scene construction outcome measures, one potential relationship was observed; a positive association between scene construction sensory details and 15 voxels in the left middle occipital cortex in the PD map (peak coordinates = -42 -87 17, peak t = 5.30, p _FWE whole brain corrected_ = 0.018). However, no corresponding negative associations with MT or R_1_, nor any corresponding relationships with R_2_^*^, were identified, even when reducing the statistical threshold to p < 0.001 uncorrected.

For future thinking, two potential associations were found. First, a positive relationship was observed in the PD map between 36 voxels in the right superior occipital gyrus and the future thinking experiential index (peak coordinates = 25 -90 33, peak t = 5.51, p _FWE whole brain corrected_ = 0.007). Reducing the statistical threshold to p < 0.001 uncorrected identified a corresponding negative association between the right superior occipital gyrus and the future thinking experiential index in the R_2_^*^ map (cluster size = 808, peak coordinates = 25 -89 35, peak t = 4.53, p _uncorrected_ < 0.001). Overall, higher water content in the right superior occipital gyrus seems to be associated with greater future thinking experiential index scores.

Second, a positive correlation was found in the R_1_ map between 5 voxels in the left middle cingulate cortex and the future thinking experiential index (peak coordinates = 0 -20 45, peak t = 5.19, p _FWE whole brain corrected_ = 0.031). Reducing the statistical threshold to p < 0.001 uncorrected identified a corresponding negative association between the left middle cingulate cortex and the future thinking experiential index in the PD map (cluster size = 28, peak coordinates = -1, -20, 45 peak t = 3.77, p _uncorrected_ < 0.001). An increase in macromolecular content and corresponding reduction in free water content in the middle cingulate cortex, may, therefore, be associated with greater future thinking experiential index scores.

Considering navigation, for the navigation movie clip recognition task, two clusters were identified on the edge of the right occipital pole in both the PD and the R_2_^*^ maps. In the PD map, these relationships were positive (Cluster 1: size = 7 voxels, peak coordinates = 32 -95 15, peak t = 5.23, p = 0.024; Cluster 2: size = 10 voxels, peak coordinates = 26 -97 20, peak t = 5.21, p = 0.026), while in the R_2_^*^ map the corresponding negative relationships were observed (Cluster 1: size = 40 voxels, peak coordinates = 32 -95 15, peak t = 5.55, p = 0.006; Cluster 2: size = 18 voxels, peak coordinates = 25 -98 19, peak t = 5.48, p = 0.009). A decrease in iron and corresponding increase in free water content in the right occipital pole, may, therefore, be related to higher navigation movie clip recognition performance.

For the navigation scene recognition task, there was a positive relationship between performance and 36 voxels in the right cuneus in the R_2_^*^ map (peak coordinates = 17 -60 10, peak t = 5.83, p _FWE whole brain corrected_ = 0.002). However, no corresponding associations in the MT saturation or PD or R_1_ maps, were identified in the right cuneus, even when reducing the statistical threshold to p < 0.001 uncorrected.

**Hippocampal ROI VBQ: significant results that were not validated across the tissue microstructure maps**

Within the scene construction sub-measures one potential relationship was identified. A negative association was found between the scene construction spatial coherence index and 77 voxels in the left hippocampus in the PD map when using the bilateral posterior hippocampal mask (peak coordinates = -19 -41 4, peak z = 3.79, p _FWE posterior hippocampus ROI corrected_ = 0.033). However, this relationship was not significant when correcting for the bilateral whole hippocampal mask (p _FWE whole hippocampus ROI corrected_ = 0.054). In addition, no corresponding associations were identified between the scene construction spatial coherence index scores and the hippocampus in the MT saturation, R_1_ or R_2_^*^ maps, even when reducing the statistical threshold to p < 0.001 uncorrected.

Within the autobiographical memory sub-measures, three potential associations were observed. First, a positive association was identified between AI emotion and a cluster of 45 voxels in the left hippocampus in the PD map when using the bilateral anterior hippocampal mask (peak coordinates = -26 -9 -27, peak z = 3.64, p _FWE anterior hippocampus ROI corrected_ = 0.033). However, this relationship was not significant when correcting for the whole hippocampus mask (p _FWE whole hippocampus ROI corrected_ = 0.073). In addition, no corresponding associations were observed in the hippocampus when using the MT saturation R_1_ or R_2_^*^maps, even when reducing the statistical threshold to p < 0.001 uncorrected.

Second, a negative association was observed between AI vividness ratings and a cluster of 37 voxels in the right hippocampus in the MT saturation map when using the bilateral whole hippocampal mask (peak coordinates = 34 -20 -12, peak z = 4.11, p _FWE whole hippocampus ROI corrected_ = 0.018), split approximately equally between the anterior and posterior hippocampal masks (anterior cluster: cluster size = 21 voxels, peak coordinates = 34 -20 -12, peak z = 4.11, p _FWE anterior hippocampus ROI corrected_ = 0.008; posterior cluster: cluster size = 16 voxels, peak coordinates = 34 -21 -12, peak z = 3.87, p _FWE posterior hippocampus ROI corrected_ = 0.027). However, no corresponding associations with the hippocampus were found in the R_1_, PD or R_2_^*^ maps, even when reducing the statistical threshold to p < 0.001 uncorrected.

AI vividness was also negatively associated with a cluster of 56 voxels in the left hippocampus in the PD map following correction for the bilateral whole hippocampal mask (peak coordinates = -28 -27 -9, peak z = 4.12, p _FWE whole hippocampus ROI corrected_ = 0.014), localised to the posterior hippocampus (p _FWE posterior hippocampus ROI corrected_ = 0.008). However, no corresponding associations were found between AI vividness and the hippocampus in the MT saturation, R_1_ or R_2_^*^ maps, even when reducing the statistical threshold to p < 0.001 uncorrected.

Within the future thinking sub-measures, two potential relationships were identified. A positive association was observed between future thinking spatial references and 33 voxels in the left hippocampus in the R_1_ map when using the bilateral posterior hippocampal mask (peak coordinates = -18 -22 -21, peak z = 3.75, p _FWE posterior hippocampus ROI corrected_ = 0.032). However, this relationship was not significant when correcting for the bilateral whole hippocampus mask (p _FWE whole hippocampus ROI corrected_ = 0.052). Furthermore, no corresponding associations were observed in the hippocampus in any of the other tissue microstructure maps, even when reducing the statistical threshold to p < 0.001 uncorrected.

Second, a negative association was found between the future thinking spatial coherence index and a cluster of 54 voxels in the right posterior hippocampus in the MT saturation map when using the bilateral posterior hippocampus mask (peak coordinates = 35, -29, -13, peak z = 3.76, p _FWE posterior hippocampus ROI corrected_ = 0.039). However, this relationship was not significant when correcting for the bilateral whole hippocampus mask (p _FWE whole hippocampus ROI corrected_ = 0.064) and the corresponding associations were not observed in the hippocampus when using any of the other tissue microstructure maps, even when reducing the statistical threshold to p < 0.001 uncorrected.

**Table S5.** Means and standard deviations of the extracted microstructure measurements for the hippocampal ROIs.

| Tissue microstructure map | Whole hippocampus | | Anterior hippocampus | | Posterior hippocampus | | Posterior/Anterior ratio | |
| --- | --- | --- | --- | --- | --- | --- | --- | --- |
|  | Mean | SD | Mean | SD | Mean | SD | Mean | SD |
|  |  |  |  |  |  |  |  |  |
| MT | 0.88 | 0.049 | 0.85 | 0.057 | 0.90 | 0.049 | 1.07 | 0.045 |
| PD | 79.87 | 1.08 | 79.60 | 1.45 | 80.09 | 1.01 | 1.01 | 0.015 |
| R_1_ | 0.65 | 0.034 | 0.63 | 0.037 | 0.66 | 0.036 | 1.04 | 0.035 |
| R_2_* | 15.42 | 0.035 | 14.76 | 1.51 | 15.97 | 1.24 | 1.09 | 0.082 |
|  |  |  |  |  |  |  |  |  |

SD: standard deviation; MT: magnetisation transfer saturation; PD: proton density; R_1_: longitudinal relaxation rate; R_2_^*^: effective transverse relaxation rate. A posterior/anterior ratio above 1 indicates greater values in the posterior relative to the anterior hippocampus.

**Table S6.** Partial correlations between task performance and hippocampal grey matter MT saturation with age, gender, total intracranial volume and MRI scanner as covariates.

| Performance variable | Whole hippocampus | | Anterior hippocampus | | Posterior hippocampus | | Posterior/Anterior hippocampus ratio | | |  |
| --- | --- | --- | --- | --- | --- | --- | --- | --- | --- | --- |
|  | r | p | r | p | r | p | | r | p | |
|  |  |  |  |  |  |  | |  |  | |
| **Scene construction** | | | | | | | | | | |
| Experiential index | -0.096 | 0.59 | -0.097 | 0.59 | -0.081 | 0.59 | | 0.043 | 0.74 | |
| Spatial references | -0.087 | 0.59 | -0.09 | 0.59 | -0.072 | 0.63 | | 0.049 | 0.73 | |
| Entities present | -0.067 | 0.66 | -0.081 | 0.59 | -0.045 | 0.73 | | 0.072 | 0.63 | |
| Sensory descriptions | -0.12 | 0.59 | -0.14 | 0.55 | -0.084 | 0.59 | | 0.11 | 0.59 | |
| Thoughts/emotions/actions | -0.05 | 0.73 | -0.081 | 0.59 | -0.014 | 0.91 | | 0.10 | 0.59 | |
| Spatial coherence index | 0.07 | 0.63 | -0.027 | 0.83 | -0.10 | 0.59 | | -0.085 | 0.59 | |
|  |  |  |  |  |  |  | |  |  | |
| **Autobiographical interview** | | | | | | | | | | |
| Internal details | -0.005 | 0.99 | 0.002 | 0.99 | -0.012 | 0.99 | | -0.026 | 0.99 | |
| Internal events | 0.009 | 0.99 | 0.012 | 0.99 | 0.005 | 0.99 | | -0.016 | 0.99 | |
| Internal time | 0.05 | 0.99 | 0.095 | 0.85 | 0.001 | 1.00 | | -0.15 | 0.75 | |
| Internal place | -0.066 | 0.93 | -0.04 | 0.99 | -0.083 | 0.88 | | -0.057 | 0.99 | |
| Internal perceptual | -0.006 | 0.99 | -0.015 | 0.99 | 0.003 | 0.99 | | 0.02 | 0.99 | |
| Internal thoughts/emotions | -0.028 | 0.99 | -0.013 | 0.99 | -0.039 | 0.99 | | -0.032 | 0.99 | |
| Vividness rating | -0.10 | 0.85 | -0.092 | 0.85 | -0.10 | 0.85 | | 0.007 | 0.99 | |
|  |  |  |  |  |  |  | |  |  | |
| **Future thinking** | | | | | | | | | | |
| Experiential index | -0.14 | 0.31 | -0.13 | 0.31 | -0.13 | 0.31 | | 0.05 | 0.81 | |
| Spatial references | -0.15 | 0.31 | -0.14 | 0.31 | -0.14 | 0.31 | | 0.05 | 0.81 | |
| Entities present | -0.038 | 0.87 | -0.032 | 0.90 | -0.038 | 0.87 | | 0.015 | 0.97 | |
| Sensory descriptions | -0.089 | 0.51 | -0.11 | 0.38 | -0.061 | 0.70 | | 0.10 | 0.38 | |
| Thoughts/emotions/actions | -0.021 | 0.94 | -0.043 | 0.87 | 0.002 | 0.99 | | 0.049 | 0.81 | |
| Spatial coherence index | -0.10 | 0.38 | -0.062 | 0.70 | -0.13 | 0.31 | | -0.062 | 0.70 | |
|  |  |  |  |  |  |  | |  |  | |
| **Navigation** | | | | | | | | | | |
| Overall navigation score | -0.11 | 0.91 | -0.071 | 0.96 | -0.14 | 0.91 | | -0.058 | 0.96 | |
| Movie clip recognition | 0.041 | 0.99 | 0.06 | 0.96 | 0.018 | 0.99 | | -0.057 | 0.96 | |
| Scene recognition | 0.07 | 0.96 | 0.071 | 0.96 | 0.059 | 0.96 | | -0.033 | 0.99 | |
| Proximity judgements | -0.076 | 0.96 | -0.054 | 0.96 | -0.087 | 0.92 | | -0.016 | 0.99 | |
| Route knowledge | -0.002 | 0.99 | 0.008 | 0.99 | -0.01 | 0.99 | | -0.033 | 0.99 | |
| Sketch map | -0.13 | 0.91 | -0.087 | 0.92 | -0.16 | 0.91 | | -0.058 | 0.96 | |
|  |  |  |  |  |  |  | |  |  | |

P values are Benjamini-Hochberg false discovery rate corrected at p < 0.05.

**Table S7.** Partial correlations between task performance and hippocampal grey matter PD with age, gender, total intracranial volume and MRI scanner as covariates.

| Performance variable | Whole hippocampus | | Anterior hippocampus | | Posterior hippocampus | | Posterior/Anterior hippocampus ratio | | |  |
| --- | --- | --- | --- | --- | --- | --- | --- | --- | --- | --- |
|  | r | p | r | p | r | p | | r | p | |
|  |  |  |  |  |  |  | |  |  | |
| **Scene construction** | | | | | | | | | | |
| Experiential index | -0.028 | 0.83 | -0.044 | 0.73 | -0.001 | 0.99 | | 0.053 | 0.72 | |
| Spatial references | -0.025 | 0.83 | -0.078 | 0.59 | 0.046 | 0.73 | | 0.13 | 0.55 | |
| Entities present | -0.083 | 0.59 | -0.085 | 0.59 | -0.06 | 0.67 | | 0.054 | 0.71 | |
| Sensory descriptions | 0.02 | 0.86 | 0.002 | 0.99 | 0.038 | 0.77 | | 0.029 | 0.82 | |
| Thoughts/emotions/actions | -0.036 | 0.77 | -0.036 | 0.77 | -0.027 | 0.83 | | 0.022 | 0.85 | |
| Spatial coherence index | -0.042 | 0.74 | -0.004 | 0.98 | -0.078 | 0.59 | | -0.06 | 0.67 | |
|  |  |  |  |  |  |  | |  |  | |
| **Autobiographical interview** | | | | | | | | | | |
| Internal details | 0.045 | 0.99 | 0.093 | 0.85 | -0.026 | 0.99 | | -0.14 | 0.75 | |
| Internal events | 0.051 | 0.99 | 0.097 | 0.85 | -0.019 | 0.99 | | -0.14 | 0.75 | |
| Internal time | -0.084 | 0.88 | -0.081 | 0.88 | -0.067 | 0.93 | | 0.039 | 0.99 | |
| Internal place | -0.005 | 0.99 | 0.00 | 1.00 | -0.011 | 0.99 | | -0.012 | 0.99 | |
| Internal perceptual | 0.025 | 0.99 | 0.048 | 0.99 | -0.01 | 0.99 | | -0.068 | 0.93 | |
| Internal thoughts/emotions | 0.066 | 0.93 | 0.12 | 0.77 | -0.018 | 0.99 | | -0.16 | 0.75 | |
| Vividness rating | -0.11 | 0.80 | -0.062 | 0.97 | -0.15 | 0.75 | | -0.048 | 0.99 | |
|  |  |  |  |  |  |  | |  |  | |
| **Future thinking** | | | | | | | | | | |
| Experiential index | -0.039 | 0.87 | -0.031 | 0.90 | -0.038 | 0.87 | | 0.005 | 0.98 | |
| Spatial references | -0.052 | 0.81 | -0.076 | 0.60 | -0.01 | 0.97 | | 0.083 | 0.55 | |
| Entities present | -0.015 | 0.97 | -0.021 | 0.94 | -0.005 | 0.98 | | 0.021 | 0.94 | |
| Sensory descriptions | 0.021 | 0.94 | 0.012 | 0.97 | 0.027 | 0.93 | | 0.007 | 0.98 | |
| Thoughts/emotions/actions | -0.042 | 0.87 | -0.003 | 0.98 | -0.079 | 0.59 | | -0.063 | 0.70 | |
| Spatial coherence index | -0.016 | 0.97 | 0.00 | 1.00 | -0.032 | 0.90 | | -0.026 | 0.93 | |
|  |  |  |  |  |  |  | |  |  | |
| **Navigation** | | | | | | | | | | |
| Overall navigation score | -0.012 | 0.99 | -0.018 | 0.99 | -0.001 | 0.99 | | 0.022 | 0.99 | |
| Movie clip recognition | -0.13 | 0.91 | -0.10 | 0.91 | -0.14 | 0.91 | | 0.012 | 0.99 | |
| Scene recognition | -0.064 | 0.96 | -0.072 | 0.96 | -0.039 | 0.99 | | 0.055 | 0.96 | |
| Proximity judgements | 0.12 | 0.91 | 0.091 | 0.91 | 0.12 | 0.91 | | -0.012 | 0.99 | |
| Route knowledge | -0.015 | 0.99 | -0.05 | 0.99 | 0.032 | 0.99 | | 0.087 | 0.92 | |
| Sketch map | -0.009 | 0.99 | -0.01 | 0.99 | -0.005 | 0.99 | | 0.009 | 0.99 | |
|  |  |  |  |  |  |  | |  |  | |

P values are Benjamini-Hochberg false discovery rate corrected at p < 0.05.

**Table S8.** Partial correlations between task performance and hippocampal grey matter R_1_ with age, gender, total intracranial volume and MRI scanner as covariates.

| Performance variable | Whole hippocampus | | Anterior hippocampus | | Posterior hippocampus | | Posterior/Anterior hippocampus ratio | | |  |
| --- | --- | --- | --- | --- | --- | --- | --- | --- | --- | --- |
|  | r | p | r | p | r | p | | r | p | |
|  |  |  |  |  |  |  | |  |  | |
| **Scene construction** | | | | | | | | | | |
| Experiential index | 0.092 | 0.59 | 0.11 | 0.59 | 0.065 | 0.67 | | -0.082 | 0.59 | |
| Spatial references | 0.11 | 0.59 | 0.13 | 0.55 | 0.087 | 0.59 | | -0.07 | 0.63 | |
| Entities present | 0.12 | 0.59 | 0.13 | 0.59 | 0.10 | 0.59 | | -0.044 | 0.73 | |
| Sensory descriptions | 0.061 | 0.67 | 0.06 | 0.67 | 0.055 | 0.71 | | -0.018 | 0.87 | |
| Thoughts/emotions/actions | 0.16 | 0.55 | 0.15 | 0.55 | 0.15 | 0.55 | | -0.001 | 0.99 | |
| Spatial coherence index | -0.039 | 0.77 | -0.035 | 0.77 | -0.035 | 0.77 | | 0.013 | 0.91 | |
|  |  |  |  |  |  |  | |  |  | |
| **Autobiographical interview** | | | | | | | | | | |
| Internal details | 0.13 | 0.77 | 0.091 | 0.85 | 0.15 | 0.75 | | 0.08 | 0.88 | |
| Internal events | 0.14 | 0.75 | 0.088 | 0.88 | 0.16 | 0.75 | | 0.11 | 0.85 | |
| Internal time | 0.072 | 0.93 | 0.077 | 0.88 | 0.061 | 0.97 | | -0.036 | 0.99 | |
| Internal place | -0.003 | 0.99 | -0.01 | 0.99 | 0.003 | 0.99 | | 0.013 | 0.99 | |
| Internal perceptual | 0.12 | 0.77 | 0.11 | 0.85 | 0.12 | 0.77 | | 0.013 | 0.99 | |
| Internal thoughts/emotions | -0.001 | 1.00 | -0.03 | 0.99 | 0.023 | 0.99 | | 0.084 | 0.88 | |
| Vividness rating | -0.034 | 0.99 | -0.038 | 0.99 | -0.027 | 0.99 | | 0.008 | 0.99 | |
|  |  |  |  |  |  |  | |  |  | |
| **Future thinking** | | | | | | | | | | |
| Experiential index | 0.13 | 0.31 | 0.12 | 0.34 | 0.13 | 0.31 | | 0.006 | 0.98 | |
| Spatial references | 0.18 | 0.31 | 0.17 | 0.31 | 0.17 | 0.31 | | -0.01 | 0.97 | |
| Entities present | 0.16 | 0.31 | 0.16 | 0.31 | 0.14 | 0.31 | | -0.033 | 0.90 | |
| Sensory descriptions | 0.11 | 0.38 | 0.11 | 0.38 | 0.098 | 0.40 | | -0.014 | 0.97 | |
| Thoughts/emotions/actions | 0.11 | 0.38 | 0.063 | 0.70 | 0.14 | 0.31 | | 0.11 | 0.38 | |
| Spatial coherence index | -0.069 | 0.70 | -0.064 | 0.70 | -0.067 | 0.70 | | 0.005 | 0.98 | |
|  |  |  |  |  |  |  | |  |  | |
| **Navigation** | | | | | | | | | | |
| Overall navigation score | -0.014 | 0.99 | 0.002 | 0.99 | -0.026 | 0.99 | | -0.045 | 0.99 | |
| Movie clip recognition | 0.021 | 0.99 | 0.056 | 0.96 | -0.011 | 0.99 | | -0.11 | 0.91 | |
| Scene recognition | 0.02 | 0.99 | 0.038 | 0.99 | 0.003 | 0.99 | | -0.055 | 0.96 | |
| Proximity judgements | -0.07 | 0.96 | -0.052 | 0.98 | -0.078 | 0.96 | | -0.033 | 0.99 | |
| Route knowledge | 0.004 | 0.99 | 0.038 | 0.99 | -0.025 | 0.99 | | -0.11 | 0.91 | |
| Sketch map | -0.015 | 0.99 | -0.004 | 0.99 | -0.023 | 0.99 | | -0.029 | 0.99 | |
|  |  |  |  |  |  |  | |  |  | |

P values are Benjamini-Hochberg false discovery rate corrected at p < 0.05.

**Table S9.** Partial correlations between task performance and hippocampal grey matter R_2_* with age, gender, total intracranial volume and MRI scanner as covariates.

| Performance variable | Whole hippocampus | | Anterior hippocampus | | Posterior hippocampus | | Posterior/Anterior hippocampus ratio | | |  |
| --- | --- | --- | --- | --- | --- | --- | --- | --- | --- | --- |
|  | r | p | r | p | r | p | | r | p | |
|  |  |  |  |  |  |  | |  |  | |
| **Scene construction** | | | | | | | | | | |
| Experiential index | 0.082 | 0.59 | 0.059 | 0.67 | 0.086 | 0.59 | | 0.004 | 0.98 | |
| Spatial references | 0.12 | 0.59 | 0.076 | 0.60 | 0.13 | 0.55 | | 0.026 | 0.83 | |
| Entities present | 0.10 | 0.59 | 0.047 | 0.73 | 0.13 | 0.55 | | 0.08 | 0.59 | |
| Sensory descriptions | 0.05 | 0.73 | 0.024 | 0.83 | 0.065 | 0.67 | | 0.047 | 0.73 | |
| Thoughts/emotions/actions | 0.081 | 0.59 | -0.005 | 0.98 | 0.15 | 0.55 | | 0.13 | 0.55 | |
| Spatial coherence index | -0.018 | 0.87 | 0.032 | 0.81 | -0.064 | 0.67 | | -0.08 | 0.59 | |
|  |  |  |  |  |  |  | |  |  | |
| **Autobiographical interview** | | | | | | | | | | |
| Internal details | 0.029 | 0.99 | 0.002 | 0.99 | 0.049 | 0.99 | | 0.034 | 0.99 | |
| Internal events | 0.028 | 0.99 | -0.004 | 0.99 | 0.055 | 0.99 | | 0.052 | 0.99 | |
| Internal time | -0.048 | 0.99 | -0.079 | 0.88 | -0.005 | 0.99 | | 0.079 | 0.88 | |
| Internal place | -0.042 | 0.99 | -0.043 | 0.99 | -0.031 | 0.99 | | 0.019 | 0.99 | |
| Internal perceptual | 0.066 | 0.93 | 0.057 | 0.99 | 0.059 | 0.98 | | -0.019 | 0.99 | |
| Internal thoughts/emotions | -0.027 | 0.99 | -0.039 | 0.99 | -0.01 | 0.99 | | 0.021 | 0.99 | |
| Vividness rating | -0.04 | 0.99 | -0.041 | 0.99 | -0.029 | 0.99 | | 0.025 | 0.99 | |
|  |  |  |  |  |  |  | |  |  | |
| **Future thinking** | | | | | | | | | | |
| Experiential index | 0.12 | 0.35 | 0.11 | 0.37 | 0.099 | 0.40 | | -0.032 | 0.90 | |
| Spatial references | 0.15 | 0.31 | 0.13 | 0.31 | 0.14 | 0.31 | | -0.014 | 0.97 | |
| Entities present | 0.13 | 0.31 | 0.13 | 0.31 | 0.095 | 0.44 | | -0.049 | 0.81 | |
| Sensory descriptions | 0.11 | 0.37 | 0.11 | 0.37 | 0.084 | 0.54 | | -0.025 | 0.94 | |
| Thoughts/emotions/actions | 0.01 | 0.97 | -0.011 | 0.97 | 0.03 | 0.92 | | 0.023 | 0.94 | |
| Spatial coherence index | 0.078 | 0.59 | 0.10 | 0.40 | 0.038 | 0.87 | | -0.061 | 0.70 | |
|  |  |  |  |  |  |  | |  |  | |
| **Navigation** | | | | | | | | | | |
| Overall navigation score | 0.065 | 0.96 | 0.018 | 0.99 | 0.097 | 0.91 | | 0.095 | 0.91 | |
| Movie clip recognition | 0.01 | 0.99 | 0.023 | 0.99 | -0.006 | 0.99 | | -0.015 | 0.99 | |
| Scene recognition | 0.099 | 0.91 | 0.067 | 0.96 | 0.11 | 0.91 | | 0.04 | 0.99 | |
| Proximity judgements | 0.017 | 0.99 | 0.032 | 0.99 | -0.002 | 0.99 | | -0.013 | 0.99 | |
| Route knowledge | 0.016 | 0.99 | -0.036 | 0.99 | 0.065 | 0.96 | | 0.11 | 0.91 | |
| Sketch map | 0.065 | 0.96 | 0.02 | 0.99 | 0.096 | 0.91 | | 0.092 | 0.91 | |
|  |  |  |  |  |  |  | |  |  | |

P values are Benjamini-Hochberg false discovery rate corrected at p < 0.05.

**Table S10.** Partial correlations between task performance and hippocampal grey matter MT saturation in the male participants with age, total intracranial volume and MRI scanner as covariates.

| Performance variable | Whole hippocampus | | Anterior hippocampus | | Posterior hippocampus | | Posterior/Anterior hippocampus ratio | | |  |
| --- | --- | --- | --- | --- | --- | --- | --- | --- | --- | --- |
|  | r | p | r | p | r | p | | r | p | |
|  |  |  |  |  |  |  | |  |  | |
| **Scene construction** | | | | | | | | | | |
| Experiential index | -0.18 | 0.70 | -0.17 | 0.70 | -0.16 | 0.70 | | 0.072 | 0.83 | |
| Spatial references | -0.075 | 0.83 | -0.094 | 0.76 | -0.041 | 0.86 | | 0.095 | 0.76 | |
| Entities present | -0.072 | 0.84 | -0.089 | 0.79 | -0.039 | 0.86 | | 0.11 | 0.76 | |
| Sensory descriptions | -0.14 | 0.70 | -0.16 | 0.70 | -0.085 | 0.81 | | 0.16 | 0.70 | |
| Thoughts/emotions/actions | -0.069 | 0.84 | -0.097 | 0.76 | -0.026 | 0.87 | | 0.10 | 0.76 | |
| Spatial coherence index | -0.21 | 0.70 | -0.12 | 0.70 | -0.26 | 0.67 | | -0.089 | 0.79 | |
|  |  |  |  |  |  |  | |  |  | |
| **Autobiographical interview** | | | | | | | | | | |
| Internal details | -0.017 | 1.00 | -0.008 | 1.00 | -0.022 | 1.00 | | -0.018 | 1.00 | |
| Internal events | -0.018 | 1.00 | -0.011 | 1.00 | -0.022 | 1.00 | | -0.008 | 1.00 | |
| Internal time | 0.10 | 0.79 | 0.16 | 0.71 | 0.027 | 1.00 | | -0.20 | 0.71 | |
| Internal place | -0.064 | 0.94 | -0.052 | 0.98 | -0.065 | 0.94 | | -0.007 | 1.00 | |
| Internal perceptual | 0.043 | 0.98 | 0.007 | 1.00 | 0.073 | 0.94 | | 0.066 | 0.94 | |
| Internal thoughts/emotions | -0.13 | 0.73 | -0.067 | 0.94 | -0.16 | 0.71 | | -0.082 | 0.90 | |
| Vividness rating | -0.24 | 0.71 | -0.21 | 0.71 | -0.23 | 0.71 | | 0.052 | 0.98 | |
|  |  |  |  |  |  |  | |  |  | |
| **Future thinking** | | | | | | | | | | |
| Experiential index | -0.22 | 0.35 | -0.18 | 0.35 | -0.21 | 0.35 | | 0.045 | 0.92 | |
| Spatial references | -0.19 | 0.35 | -0.16 | 0.35 | -0.20 | 0.35 | | 0.029 | 0.92 | |
| Entities present | -0.076 | 0.84 | -0.055 | 0.87 | -0.084 | 0.78 | | 0.02 | 0.93 | |
| Sensory descriptions | -0.054 | 0.87 | -0.071 | 0.85 | -0.026 | 0.92 | | 0.12 | 0.52 | |
| Thoughts/emotions/actions | -0.15 | 0.35 | -0.14 | 0.42 | -0.14 | 0.41 | | 0.036 | 0.92 | |
| Spatial coherence index | 0.20 | 0.35 | -0.14 | 0.42 | -0.23 | 0.35 | | -0.029 | 0.92 | |
|  |  |  |  |  |  |  | |  |  | |
| **Navigation** | | | | | | | | | | |
| Overall navigation score | -0.092 | 0.95 | -0.057 | 0.95 | -0.11 | 0.95 | | -0.039 | 0.95 | |
| Movie clip recognition | 0.00 | 1.00 | 0.037 | 0.95 | -0.039 | 0.95 | | -0.079 | 0.95 | |
| Scene recognition | 0.11 | 0.95 | 0.11 | 0.95 | 0.087 | 0.95 | | -0.066 | 0.95 | |
| Proximity judgements | -0.014 | 0.95 | 0.002 | 1.00 | -0.029 | 0.95 | | -0.018 | 0.95 | |
| Route knowledge | 0.017 | 0.95 | -0.021 | 0.95 | 0.054 | 0.95 | | 0.083 | 0.95 | |
| Sketch map | -0.12 | 0.95 | -0.071 | 0.95 | -0.14 | 0.95 | | -0.052 | 0.95 | |
|  |  |  |  |  |  |  | |  |  | |

P values are Benjamini-Hochberg false discovery rate corrected at p < 0.05.

**Table S11.** Partial correlations between task performance and hippocampal grey matter PD in the male participants with age, total intracranial volume and MRI scanner as covariates.

| Performance variable | Whole hippocampus | | Anterior hippocampus | | Posterior hippocampus | | Posterior/Anterior hippocampus ratio | | |  |
| --- | --- | --- | --- | --- | --- | --- | --- | --- | --- | --- |
|  | r | p | r | p | r | p | | r | p | |
|  |  |  |  |  |  |  | |  |  | |
| **Scene construction** | | | | | | | | | | |
| Experiential index | 0.073 | 0.83 | 0.034 | 0.86 | 0.098 | 0.76 | | 0.038 | 0.86 | |
| Spatial references | 0.045 | 0.85 | -0.055 | 0.85 | 0.15 | 0.70 | | 0.18 | 0.70 | |
| Entities present | 0.005 | 0.97 | -0.018 | 0.92 | 0.031 | 0.86 | | 0.046 | 0.85 | |
| Sensory descriptions | -0.046 | 0.85 | -0.046 | 0.85 | -0.032 | 0.86 | | 0.027 | 0.87 | |
| Thoughts/emotions/actions | 0.13 | 0.70 | 0.099 | 0.76 | 0.12 | 0.70 | | -0.019 | 0.92 | |
| Spatial coherence index | -0.052 | 0.85 | -0.03 | 0.86 | -0.062 | 0.85 | | -0.015 | 0.93 | |
|  |  |  |  |  |  |  | |  |  | |
| **Autobiographical interview** | | | | | | | | | | |
| Internal details | 0.086 | 0.88 | 0.14 | 0.71 | -0.007 | 1.00 | | -0.16 | 0.71 | |
| Internal events | 0.088 | 0.86 | 0.14 | 0.71 | 0.00 | 1.00 | | -0.16 | 0.71 | |
| Internal time | -0.047 | 0.98 | 0.016 | 1.00 | -0.11 | 0.79 | | -0.10 | 0.80 | |
| Internal place | 0.063 | 0.94 | 0.044 | 0.98 | 0.067 | 0.94 | | 0.001 | 1.00 | |
| Internal perceptual | 0.065 | 0.94 | 0.089 | 0.86 | 0.017 | 1.00 | | -0.087 | 0.87 | |
| Internal thoughts/emotions | 0.05 | 0.98 | 0.11 | 0.79 | -0.032 | 1.00 | | -0.14 | 0.71 | |
| Vividness rating | -0.13 | 0.73 | -0.077 | 0.92 | -0.16 | 0.71 | | -0.04 | 1.00 | |
|  |  |  |  |  |  |  | |  |  | |
| **Future thinking** | | | | | | | | | | |
| Experiential index | 0.033 | 0.92 | 0.056 | 0.87 | -0.004 | 0.97 | | -0.066 | 0.86 | |
| Spatial references | -0.028 | 0.92 | -0.052 | 0.89 | 0.01 | 0.94 | | 0.063 | 0.86 | |
| Entities present | 0.063 | 0.86 | 0.037 | 0.92 | 0.074 | 0.85 | | 0.017 | 0.93 | |
| Sensory descriptions | -0.038 | 0.92 | -0.009 | 0.94 | -0.06 | 0.86 | | -0.036 | 0.92 | |
| Thoughts/emotions/actions | 0.07 | 0.85 | 0.13 | 0.46 | -0.022 | 0.93 | | -0.16 | 0.35 | |
| Spatial coherence index | 0.024 | 0.93 | 0.01 | 0.94 | 0.033 | 0.92 | | 0.014 | 0.93 | |
|  |  |  |  |  |  |  | |  |  | |
| **Navigation** | | | | | | | | | | |
| Overall navigation score | 0.08 | 0.95 | 0.11 | 0.95 | 0.02 | 0.95 | | -0.10 | 0.95 | |
| Movie clip recognition | -0.17 | 0.95 | -0.12 | 0.95 | -0.19 | 0.95 | | -0.012 | 0.95 | |
| Scene recognition | 0.013 | 0.95 | 0.052 | 0.95 | -0.039 | 0.95 | | -0.086 | 0.95 | |
| Proximity judgements | 0.18 | 0.95 | 0.12 | 0.95 | 0.20 | 0.95 | | 0.024 | 0.95 | |
| Route knowledge | 0.081 | 0.95 | 0.051 | 0.95 | 0.093 | 0.95 | | 0.016 | 0.95 | |
| Sketch map | 0.077 | 0.95 | 0.11 | 0.95 | 0.008 | 0.97 | | -0.12 | 0.95 | |
|  |  |  |  |  |  |  | |  |  | |

P values are Benjamini-Hochberg false discovery rate corrected at p < 0.05.

**Table S12.** Partial correlations between task performance and hippocampal grey matter R_1_ in the male participants with age, total intracranial volume and MRI scanner as covariates.

| Performance variable | Whole hippocampus | | Anterior hippocampus | | Posterior hippocampus | | Posterior/Anterior hippocampus ratio | | |  |
| --- | --- | --- | --- | --- | --- | --- | --- | --- | --- | --- |
|  | r | p | r | p | r | p | | r | p | |
|  |  |  |  |  |  |  | |  |  | |
| **Scene construction** | | | | | | | | | | |
| Experiential index | 0.055 | 0.85 | 0.072 | 0.83 | 0.034 | 0.86 | | -0.059 | 0.85 | |
| Spatial references | 0.083 | 0.82 | 0.13 | 0.70 | 0.039 | 0.86 | | -0.13 | 0.70 | |
| Entities present | 0.08 | 0.83 | 0.099 | 0.76 | 0.055 | 0.85 | | -0.066 | 0.84 | |
| Sensory descriptions | 0.13 | 0.70 | 0.13 | 0.70 | 0.12 | 0.70 | | -0.03 | 0.86 | |
| Thoughts/emotions/actions | 0.15 | 0.70 | 0.094 | 0.76 | 0.17 | 0.70 | | 0.11 | 0.73 | |
| Spatial coherence index | -0.053 | 0.85 | -0.051 | 0.85 | -0.049 | 0.85 | | 0.014 | 0.93 | |
|  |  |  |  |  |  |  | |  |  | |
| **Autobiographical interview** | | | | | | | | | | |
| Internal details | 0.18 | 0.71 | 0.14 | 0.71 | 0.19 | 0.71 | | 0.06 | 0.95 | |
| Internal events | 0.17 | 0.71 | 0.14 | 0.73 | 0.18 | 0.71 | | 0.056 | 0.98 | |
| Internal time | 0.15 | 0.71 | 0.12 | 0.75 | 0.15 | 0.71 | | 0.044 | 0.98 | |
| Internal place | 0.11 | 0.79 | 0.12 | 0.75 | 0.093 | 0.85 | | -0.051 | 0.98 | |
| Internal perceptual | 0.18 | 0.71 | 0.16 | 0.71 | 0.17 | 0.71 | | 0.00 | 1.00 | |
| Internal thoughts/emotions | -0.022 | 1.00 | -0.065 | 0.94 | 0.016 | 1.00 | | 0.12 | 0.73 | |
| Vividness rating | -0.002 | 1.00 | -0.017 | 1.00 | 0.01 | 1.00 | | 0.026 | 1.00 | |
|  |  |  |  |  |  |  | |  |  | |
| **Future thinking** | | | | | | | | | | |
| Experiential index | 0.16 | 0.35 | 0.12 | 0.48 | 0.17 | 0.35 | | 0.063 | 0.86 | |
| Spatial references | 0.17 | 0.35 | 0.15 | 0.37 | 0.17 | 0.35 | | 0.024 | 0.93 | |
| Entities present | 0.14 | 0.42 | 0.16 | 0.35 | 0.11 | 0.57 | | -0.072 | 0.85 | |
| Sensory descriptions | 0.19 | 0.35 | 0.17 | 0.35 | 0.19 | 0.35 | | 0.028 | 0.92 | |
| Thoughts/emotions/actions | 0.11 | 0.54 | 0.018 | 0.93 | 0.18 | 0.35 | | 0.23 | 0.35 | |
| Spatial coherence index | -0.051 | 0.89 | -0.038 | 0.92 | -0.056 | 0.87 | | -0.015 | 0.93 | |
|  |  |  |  |  |  |  | |  |  | |
| **Navigation** | | | | | | | | | | |
| Overall navigation score | -0.034 | 0.95 | -0.043 | 0.95 | -0.022 | 0.95 | | 0.029 | 0.95 | |
| Movie clip recognition | -0.012 | 0.95 | 0.017 | 0.95 | -0.035 | 0.95 | | -0.075 | 0.95 | |
| Scene recognition | 0.028 | 0.95 | 0.017 | 0.95 | 0.035 | 0.95 | | 0.027 | 0.95 | |
| Proximity judgements | 0.033 | 0.95 | 0.042 | 0.95 | 0.022 | 0.95 | | -0.02 | 0.95 | |
| Route knowledge | 0.033 | 0.95 | 0.049 | 0.95 | 0.017 | 0.95 | | -0.052 | 0.95 | |
| Sketch map | -0.047 | 0.95 | -0.061 | 0.95 | -0.031 | 0.95 | | 0.043 | 0.95 | |
|  |  |  |  |  |  |  | |  |  | |

P values are Benjamini-Hochberg false discovery rate corrected at p < 0.05.

**Table S13.** Partial correlations between task performance and hippocampal grey matter R_2_* in the male participants with age, total intracranial volume and MRI scanner as covariates.

| Performance variable | Whole hippocampus | | Anterior hippocampus | | Posterior hippocampus | | Posterior/Anterior hippocampus ratio | | |  |
| --- | --- | --- | --- | --- | --- | --- | --- | --- | --- | --- |
|  | r | p | r | p | r | p | | r | p | |
|  |  |  |  |  |  |  | |  |  | |
| **Scene construction** | | | | | | | | | | |
| Experiential index | 0.13 | 0.70 | 0.079 | 0.83 | 0.15 | 0.70 | | 0.034 | 0.86 | |
| Spatial references | 0.17 | 0.70 | 0.14 | 0.70 | 0.15 | 0.70 | | -0.046 | 0.85 | |
| Entities present | 0.16 | 0.70 | 0.12 | 0.70 | 0.16 | 0.70 | | 0.005 | 0.97 | |
| Sensory descriptions | 0.12 | 0.70 | 0.069 | 0.84 | 0.15 | 0.70 | | 0.052 | 0.85 | |
| Thoughts/emotions/actions | 0.12 | 0.70 | 0.011 | 0.94 | 0.20 | 0.70 | | 0.14 | 0.70 | |
| Spatial coherence index | 0.018 | 0.92 | -0.003 | 0.98 | 0.037 | 0.86 | | 0.067 | 0.84 | |
|  |  |  |  |  |  |  | |  |  | |
| **Autobiographical interview** | | | | | | | | | | |
| Internal details | 0.14 | 0.71 | 0.098 | 0.84 | 0.15 | 0.71 | | -0.002 | 1.00 | |
| Internal events | 0.13 | 0.73 | 0.076 | 0.92 | 0.15 | 0.71 | | 0.036 | 1.00 | |
| Internal time | 0.006 | 1.00 | 0.013 | 1.00 | -0.004 | 1.00 | | -0.029 | 1.00 | |
| Internal place | 0.10 | 0.79 | 0.062 | 0.94 | 0.12 | 0.73 | | 0.016 | 1.00 | |
| Internal perceptual | 0.14 | 0.71 | 0.13 | 0.73 | 0.11 | 0.78 | | -0.066 | 0.94 | |
| Internal thoughts/emotions | 0.033 | 1.00 | 0.011 | 1.00 | 0.049 | 0.98 | | 0.021 | 1.00 | |
| Vividness rating | -0.011 | 1.00 | -0.095 | 0.84 | 0.089 | 0.86 | | 0.20 | 0.71 | |
|  |  |  |  |  |  |  | |  |  | |
| **Future thinking** | | | | | | | | | | |
| Experiential index | 0.18 | 0.35 | 0.14 | 0.42 | 0.18 | 0.35 | | -0.011 | 0.94 | |
| Spatial references | 0.18 | 0.35 | 0.11 | 0.53 | 0.21 | 0.35 | | 0.046 | 0.92 | |
| Entities present | 0.21 | 0.35 | 0.21 | 0.35 | 0.15 | 0.35 | | -0.096 | 0.66 | |
| Sensory descriptions | 0.19 | 0.35 | 0.18 | 0.35 | 0.16 | 0.35 | | -0.033 | 0.92 | |
| Thoughts/emotions/actions | 0.12 | 0.51 | 0.061 | 0.86 | 0.15 | 0.35 | | 0.026 | 0.92 | |
| Spatial coherence index | 0.17 | 0.35 | 0.13 | 0.47 | 0.17 | 0.35 | | 0.018 | 0.93 | |
|  |  |  |  |  |  |  | |  |  | |
| **Navigation** | | | | | | | | | | |
| Overall navigation score | -0.006 | 0.97 | -0.062 | 0.95 | 0.06 | 0.95 | | 0.14 | 0.95 | |
| Movie clip recognition | -0.15 | 0.95 | -0.091 | 0.95 | -0.18 | 0.95 | | -0.026 | 0.95 | |
| Scene recognition | 0.12 | 0.95 | 0.044 | 0.95 | 0.18 | 0.95 | | 0.11 | 0.95 | |
| Proximity judgements | 0.012 | 0.95 | -0.021 | 0.95 | 0.045 | 0.95 | | 0.096 | 0.95 | |
| Route knowledge | 0.066 | 0.95 | -0.022 | 0.95 | 0.15 | 0.95 | | 0.16 | 0.95 | |
| Sketch map | -0.02 | 0.95 | -0.068 | 0.95 | 0.041 | 0.95 | | 0.13 | 0.95 | |
|  |  |  |  |  |  |  | |  |  | |

P values are Benjamini-Hochberg false discovery rate corrected at p < 0.05.

**Table S14.** Partial correlations between tasks performance and hippocampal grey matter MT saturation in the female participants with age, total intracranial volume and MRI scanner as covariates.

| Performance variable | Whole hippocampus | | Anterior hippocampus | | Posterior hippocampus | | Posterior/Anterior hippocampus ratio | | |  |
| --- | --- | --- | --- | --- | --- | --- | --- | --- | --- | --- |
|  | r | p | r | p | r | p | | r | p | |
|  |  |  |  |  |  |  | |  |  | |
| **Scene construction** | | | | | | | | | | |
| Experiential index | -0.002 | 1.00 | -0.005 | 1.00 | 0.001 | 1.00 | | -0.001 | 1.00 | |
| Spatial references | -0.076 | 0.95 | -0.070 | 0.96 | -0.074 | 0.95 | | 0.004 | 1.00 | |
| Entities present | -0.048 | 0.96 | -0.059 | 0.96 | -0.034 | 0.96 | | 0.035 | 0.96 | |
| Sensory descriptions | -0.084 | 0.94 | -0.093 | 0.88 | -0.067 | 0.96 | | 0.034 | 0.96 | |
| Thoughts/emotions/actions | -0.002 | 1.00 | -0.034 | 0.96 | 0.027 | 0.96 | | 0.095 | 0.88 | |
| Spatial coherence index | 0.065 | 0.96 | 0.081 | 0.95 | 0.044 | 0.96 | | -0.089 | 0.91 | |
|  |  |  |  |  |  |  | |  |  | |
| **Autobiographical interview** | | | | | | | | | | |
| Internal details | 0.010 | 1.00 | 0.012 | 1.00 | 0.007 | 1.00 | | -0.023 | 1.00 | |
| Internal events | 0.021 | 1.00 | 0.022 | 1.00 | 0.018 | 1.00 | | -0.020 | 1.00 | |
| Internal time | 0.020 | 1.00 | 0.051 | 1.00 | -0.009 | 1.00 | | -0.099 | 1.00 | |
| Internal place | -0.078 | 1.00 | -0.050 | 1.00 | -0.095 | 1.00 | | -0.069 | 1.00 | |
| Internal perceptual | -0.033 | 1.00 | -0.030 | 1.00 | -0.032 | 1.00 | | -0.009 | 1.00 | |
| Internal thoughts/emotions | 0.084 | 1.00 | 0.062 | 1.00 | 0.096 | 1.00 | | 0.033 | 1.00 | |
| Vividness rating | -0.011 | 1.00 | 0.000 | 1.00 | -0.019 | 1.00 | | -0.044 | 1.00 | |
|  |  |  |  |  |  |  | |  |  | |
| **Future thinking** | | | | | | | | | | |
| Experiential index | -0.035 | 0.97 | -0.054 | 0.89 | -0.015 | 1.00 | | 0.054 | 0.89 | |
| Spatial references | -0.11 | 0.81 | -0.123 | 0.81 | -0.094 | 0.81 | | 0.062 | 0.89 | |
| Entities present | 0.001 | 1.00 | -0.009 | 1.00 | 0.010 | 1.00 | | 0.018 | 1.00 | |
| Sensory descriptions | -0.13 | 0.81 | -0.146 | 0.81 | -0.104 | 0.81 | | 0.076 | 0.83 | |
| Thoughts/emotions/actions | 0.13 | 0.81 | 0.087 | 0.81 | 0.151 | 0.81 | | 0.059 | 0.89 | |
| Spatial coherence index | 0.019 | 1.00 | 0.047 | 0.93 | -0.009 | 1.00 | | -0.104 | 0.81 | |
|  |  |  |  |  |  |  | |  |  | |
| **Navigation** | | | | | | | | | | |
| Overall navigation score | -0.10 | 0.71 | -0.059 | 0.88 | -0.13 | 0.65 | | -0.081 | 0.80 | |
| Movie clip recognition | 0.087 | 0.79 | 0.091 | 0.76 | 0.075 | 0.82 | | -0.029 | 0.91 | |
| Scene recognition | 0.047 | 0.91 | 0.042 | 0.91 | 0.047 | 0.91 | | -0.001 | 0.99 | |
| Proximity judgements | -0.13 | 0.65 | -0.12 | 0.71 | -0.13 | 0.65 | | 0.008 | 0.97 | |
| Route knowledge | 0.013 | 0.96 | 0.071 | 0.82 | -0.040 | 0.91 | | -0.18 | 0.65 | |
| Sketch map | -0.12 | 0.71 | -0.078 | 0.82 | -0.15 | 0.65 | | -0.066 | 0.85 | |
|  |  |  |  |  |  |  | |  |  | |

P values are Benjamini-Hochberg false discovery rate corrected at p < 0.05.

**Table S15.** Partial correlations between tasks performance and hippocampal grey matter PD in the female participants with age, total intracranial volume and MRI scanner as covariates.

| Performance variable | Whole hippocampus | | Anterior hippocampus | | Posterior hippocampus | | Posterior/Anterior hippocampus ratio | | |  |
| --- | --- | --- | --- | --- | --- | --- | --- | --- | --- | --- |
|  | r | p | r | p | r | p | | r | p | |
|  |  |  |  |  |  |  | |  |  | |
| **Scene construction** | | | | | | | | | | |
| Experiential index | -0.12 | 0.88 | -0.12 | 0.88 | -0.11 | 0.88 | | 0.058 | 0.96 | |
| Spatial references | -0.080 | 0.95 | -0.098 | 0.88 | -0.044 | 0.96 | | 0.094 | 0.88 | |
| Entities present | -0.16 | 0.77 | -0.14 | 0.77 | -0.15 | 0.77 | | 0.060 | 0.96 | |
| Sensory descriptions | 0.095 | 0.88 | 0.066 | 0.96 | 0.11 | 0.88 | | 0.012 | 0.98 | |
| Thoughts/emotions/actions | -0.16 | 0.77 | -0.14 | 0.77 | -0.16 | 0.77 | | 0.049 | 0.96 | |
| Spatial coherence index | -0.031 | 0.96 | 0.029 | 0.96 | -0.098 | 0.88 | | -0.13 | 0.83 | |
|  |  |  |  |  |  |  | |  |  | |
| **Autobiographical interview** | | | | | | | | | | |
| Internal details | 0.005 | 1.00 | 0.037 | 1.00 | -0.036 | 1.00 | | -0.089 | 1.00 | |
| Internal events | 0.013 | 1.00 | 0.051 | 1.00 | -0.035 | 1.00 | | -0.11 | 1.00 | |
| Internal time | -0.11 | 1.00 | -0.16 | 1.00 | -0.033 | 1.00 | | 0.19 | 1.00 | |
| Internal place | -0.074 | 1.00 | -0.077 | 1.00 | -0.057 | 1.00 | | 0.048 | 1.00 | |
| Internal perceptual | -0.008 | 1.00 | 0.003 | 1.00 | -0.021 | 1.00 | | -0.026 | 1.00 | |
| Internal thoughts/emotions | 0.084 | 1.00 | 0.14 | 1.00 | 0.002 | 1.00 | | -0.19 | 1.00 | |
| Vividness rating | -0.095 | 1.00 | -0.045 | 1.00 | -0.14 | 1.00 | | -0.072 | 1.00 | |
|  |  |  |  |  |  |  | |  |  | |
| **Future thinking** | | | | | | | | | | |
| Experiential index | -0.13 | 0.81 | -0.14 | 0.81 | -0.090 | 0.81 | | 0.11 | 0.81 | |
| Spatial references | -0.068 | 0.87 | -0.086 | 0.81 | -0.033 | 0.98 | | 0.088 | 0.81 | |
| Entities present | -0.12 | 0.81 | -0.11 | 0.81 | -0.11 | 0.81 | | 0.046 | 0.93 | |
| Sensory descriptions | 0.096 | 0.81 | 0.052 | 0.89 | 0.13 | 0.81 | | 0.052 | 0.89 | |
| Thoughts/emotions/actions | -0.15 | 0.81 | -0.13 | 0.81 | -0.14 | 0.81 | | 0.040 | 0.94 | |
| Spatial coherence index | -0.059 | 0.89 | -0.012 | 1.00 | -0.11 | 0.81 | | -0.085 | 0.81 | |
|  |  |  |  |  |  |  | |  |  | |
| **Navigation** | | | | | | | | | | |
| Overall navigation score | -0.10 | 0.71 | -0.16 | 0.65 | -0.022 | 0.92 | | 0.19 | 0.65 | |
| Movie clip recognition | -0.11 | 0.71 | -0.11 | 0.71 | -0.096 | 0.75 | | 0.058 | 0.88 | |
| Scene recognition | -0.13 | 0.65 | -0.18 | 0.65 | -0.044 | 0.91 | | 0.21 | 0.65 | |
| Proximity judgements | 0.049 | 0.91 | 0.042 | 0.91 | 0.050 | 0.91 | | -0.011 | 0.96 | |
| Route knowledge | -0.10 | 0.71 | -0.15 | 0.65 | -0.032 | 0.91 | | 0.17 | 0.65 | |
| Sketch map | -0.093 | 0.76 | -0.14 | 0.65 | -0.017 | 0.95 | | 0.18 | 0.65 | |
|  |  |  |  |  |  |  | |  |  | |

P values are Benjamini-Hochberg false discovery rate corrected at p < 0.05.

**Table S16.** Partial correlations between tasks performance and hippocampal grey matter R_1_ in the female participants with age, total intracranial volume and MRI scanner as covariates.

| Performance variable | Whole hippocampus | | Anterior hippocampus | | Posterior hippocampus | | Posterior/Anterior hippocampus ratio | | |  |
| --- | --- | --- | --- | --- | --- | --- | --- | --- | --- | --- |
|  | r | p | r | p | r | p | | r | p | |
|  |  |  |  |  |  |  | |  |  | |
| **Scene construction** | | | | | | | | | | |
| Experiential index | 0.13 | 0.88 | 0.15 | 0.77 | 0.094 | 0.88 | | -0.10 | 0.88 | |
| Spatial references | 0.15 | 0.77 | 0.15 | 0.77 | 0.14 | 0.77 | | -0.019 | 0.96 | |
| Entities present | 0.16 | 0.77 | 0.15 | 0.77 | 0.14 | 0.77 | | -0.024 | 0.96 | |
| Sensory descriptions | -0.017 | 0.96 | -0.021 | 0.96 | -0.012 | 0.98 | | 0.007 | 1.00 | |
| Thoughts/emotions/actions | 0.18 | 0.77 | 0.20 | 0.77 | 0.145 | 0.77 | | -0.094 | 0.88 | |
| Spatial coherence index | -0.033 | 0.96 | -0.040 | 0.96 | -0.025 | 0.96 | | 0.021 | 0.96 | |
|  |  |  |  |  |  |  | |  |  | |
| **Autobiographical interview** | | | | | | | | | | |
| Internal details | 0.11 | 1.00 | 0.070 | 1.00 | 0.13 | 1.00 | | 0.084 | 1.00 | |
| Internal events | 0.11 | 1.00 | 0.045 | 1.00 | 0.15 | 1.00 | | 0.16 | 1.00 | |
| Internal time | -0.010 | 1.00 | 0.022 | 1.00 | -0.037 | 1.00 | | -0.10 | 1.00 | |
| Internal place | -0.022 | 1.00 | -0.027 | 1.00 | -0.016 | 1.00 | | 0.014 | 1.00 | |
| Internal perceptual | 0.10 | 1.00 | 0.093 | 1.00 | 0.10 | 1.00 | | 0.001 | 1.00 | |
| Internal thoughts/emotions | 0.032 | 1.00 | 0.019 | 1.00 | 0.041 | 1.00 | | 0.033 | 1.00 | |
| Vividness rating | -0.082 | 1.00 | -0.080 | 1.00 | -0.077 | 1.00 | | 0.000 | 1.00 | |
|  |  |  |  |  |  |  | |  |  | |
| **Future thinking** | | | | | | | | | | |
| Experiential index | 0.12 | 0.81 | 0.13 | 0.81 | 0.094 | 0.81 | | -0.064 | 0.89 | |
| Spatial references | 0.16 | 0.81 | 0.16 | 0.81 | 0.14 | 0.81 | | -0.030 | 0.99 | |
| Entities present | 0.21 | 0.81 | 0.20 | 0.81 | 0.21 | 0.81 | | 0.006 | 1.00 | |
| Sensory descriptions | -0.002 | 1.00 | 0.015 | 1.00 | -0.016 | 1.00 | | -0.054 | 0.89 | |
| Thoughts/emotions/actions | 0.13 | 0.81 | 0.12 | 0.81 | 0.12 | 0.81 | | 0.006 | 1.00 | |
| Spatial coherence index | -0.072 | 0.85 | -0.077 | 0.83 | -0.062 | 0.89 | | 0.025 | 1.00 | |
|  |  |  |  |  |  |  | |  |  | |
| **Navigation** | | | | | | | | | | |
| Overall navigation score | 0.027 | 0.92 | 0.071 | 0.82 | -0.012 | 0.96 | | -0.14 | 0.65 | |
| Movie clip recognition | 0.069 | 0.83 | 0.12 | 0.71 | 0.024 | 0.92 | | -0.15 | 0.65 | |
| Scene recognition | 0.034 | 0.91 | 0.076 | 0.82 | -0.004 | 0.99 | | -0.13 | 0.65 | |
| Proximity judgements | -0.13 | 0.65 | -0.099 | 0.73 | -0.15 | 0.65 | | -0.081 | 0.80 | |
| Route knowledge | -0.018 | 0.95 | 0.034 | 0.91 | -0.059 | 0.88 | | -0.16 | 0.65 | |
| Sketch map | 0.035 | 0.91 | 0.072 | 0.82 | 0.001 | 0.99 | | -0.11 | 0.71 | |
|  |  |  |  |  |  |  | |  |  | |

P values are Benjamini-Hochberg false discovery rate corrected at p < 0.05.

**Table S17.** Partial correlations between tasks performance and hippocampal grey matter R_2_* in the female participants with age, total intracranial volume and MRI scanner as covariates.

| Performance variable | Whole hippocampus | | Anterior hippocampus | | Posterior hippocampus | | Posterior/Anterior hippocampus ratio | | |  |
| --- | --- | --- | --- | --- | --- | --- | --- | --- | --- | --- |
|  | r | p | r | p | r | p | | r | p | |
|  |  |  |  |  |  |  | |  |  | |
| **Scene construction** | | | | | | | | | | |
| Experiential index | 0.026 | 0.96 | 0.035 | 0.96 | 0.013 | 0.98 | | -0.047 | 0.96 | |
| Spatial references | 0.075 | 0.95 | 0.025 | 0.96 | 0.11 | 0.88 | | 0.074 | 0.95 | |
| Entities present | 0.050 | 0.96 | -0.018 | 0.96 | 0.10 | 0.88 | | 0.14 | 0.77 | |
| Sensory descriptions | -0.034 | 0.96 | -0.030 | 0.96 | -0.032 | 0.96 | | 0.017 | 0.96 | |
| Thoughts/emotions/actions | 0.041 | 0.96 | -0.018 | 0.96 | 0.088 | 0.91 | | 0.096 | 0.88 | |
| Spatial coherence index | -0.059 | 0.96 | 0.067 | 0.96 | -0.17 | 0.77 | | -0.27 | 0.55 | |
|  |  |  |  |  |  |  | |  |  | |
| **Autobiographical interview** | | | | | | | | | | |
| Internal details | -0.066 | 1.00 | -0.090 | 1.00 | -0.032 | 1.00 | | 0.065 | 1.00 | |
| Internal events | -0.062 | 1.00 | -0.092 | 1.00 | -0.022 | 1.00 | | 0.085 | 1.00 | |
| Internal time | -0.11 | 1.00 | -0.19 | 1.00 | -0.024 | 1.00 | | 0.12 | 1.00 | |
| Internal place | -0.12 | 1.00 | -0.11 | 1.00 | -0.11 | 1.00 | | 0.025 | 1.00 | |
| Internal perceptual | 0.019 | 1.00 | 0.015 | 1.00 | 0.020 | 1.00 | | 0.001 | 1.00 | |
| Internal thoughts/emotions | -0.087 | 1.00 | -0.084 | 1.00 | -0.073 | 1.00 | | -0.006 | 1.00 | |
| Vividness rating | -0.065 | 1.00 | 0.001 | 1.00 | -0.12 | 1.00 | | -0.14 | 1.00 | |
|  |  |  |  |  |  |  | |  |  | |
| **Future thinking** | | | | | | | | | | |
| Experiential index | 0.042 | 0.94 | 0.076 | 0.83 | 0.003 | 1.00 | | -0.082 | 0.82 | |
| Spatial references | 0.11 | 0.81 | 0.14 | 0.81 | 0.069 | 0.87 | | -0.091 | 0.81 | |
| Entities present | 0.041 | 0.94 | 0.029 | 0.99 | 0.043 | 0.94 | | 0.023 | 1.00 | |
| Sensory descriptions | 0.011 | 1.00 | 0.020 | 1.00 | 0.00 | 1.00 | | -0.013 | 1.00 | |
| Thoughts/emotions/actions | -0.10 | 0.81 | -0.087 | 0.81 | -0.094 | 0.81 | | -0.003 | 1.00 | |
| Spatial coherence index | -0.014 | 1.00 | 0.077 | 0.83 | -0.097 | 0.81 | | -0.19 | 0.81 | |
|  |  |  |  |  |  |  | |  |  | |
| **Navigation** | | | | | | | | | | |
| Overall navigation score | 0.13 | 0.65 | 0.12 | 0.71 | 0.12 | 0.71 | | 0.006 | 0.98 | |
| Movie clip recognition | 0.13 | 0.65 | 0.13 | 0.65 | 0.11 | 0.71 | | -0.029 | 0.91 | |
| Scene recognition | 0.085 | 0.79 | 0.091 | 0.76 | 0.064 | 0.85 | | -0.029 | 0.91 | |
| Proximity judgements | 0.039 | 0.91 | 0.11 | 0.71 | -0.033 | 0.91 | | -0.15 | 0.65 | |
| Route knowledge | -0.043 | 0.91 | -0.048 | 0.91 | -0.031 | 0.91 | | 0.024 | 0.92 | |
| Sketch map | 0.15 | 0.65 | 0.13 | 0.65 | 0.13 | 0.65 | | 0.014 | 0.96 | |
|  |  |  |  |  |  |  | |  |  | |

P values are Benjamini-Hochberg false discovery rate corrected at p < 0.05.

**Table S18.** Details of the groups created for each task when dividing by median performance.

| Performance variable | Median performance | N (low performance) | N (high performance) |
| --- | --- | --- | --- |
|  |  |  |  |
| **Scene construction** |  |  |  |
| Experiential index | 41.2 | 109 | 108 |
| Spatial references | 3.29 | 112 | 105 |
| Entities present | 9.71 | 112 | 105 |
| Sensory descriptions | 12.14 | 112 | 105 |
| Thoughts/emotions/actions | 3.14 | 114 | 103 |
| Spatial coherence index | 3.0 | 112 | 105 |
|  |  |  |  |
| **Autobiographical interview** | | | |
| Internal details | 23.2 | 110 | 107 |
| Internal events | 10.6 | 111 | 106 |
| Internal time | 1.4 | 128 | 89 |
| Internal place | 2.2 | 123 | 94 |
| Internal perceptual | 5.4 | 109 | 108 |
| Internal thoughts/emotions | 3.2 | 111 | 106 |
| Vividness rating | 4.6 | 114 | 103 |
|  |  |  |  |
| **Future thinking** |  |  |  |
| Experiential index | 39.8 | 111 | 106 |
| Spatial references | 2.3 | 123 | 94 |
| Entities present | 10.0 | 114 | 103 |
| Sensory descriptions | 8.67 | 117 | 100 |
| Thoughts/emotions/actions | 5.0 | 116 | 101 |
| Spatial coherence index | 2.67 | 115 | 102 |
|  |  |  |  |
| **Navigation** |  |  |  |
| Overall navigation score | 144.0 | 109 | 108 |
| Scene recognition | 30.0 | 146 | 71 |
| Proximity judgements | 8.0 | 159 | 58 |
| Route knowledge | 11.0 | 114 | 103 |
| Sketch map | 81 | 111 | 106 |
|  |  |  |  |

**Table S19.** Comparison of hippocampal grey matter MT saturation when dividing the sample into two groups determined by their median performance on each cognitive task, with age, gender, total intracranial volume and MRI scanner included as covariates.

| Performance variable | Whole hippocampus | | Anterior hippocampus | | Posterior hippocampus | | Posterior/Anterior hippocampus ratio | | |  |
| --- | --- | --- | --- | --- | --- | --- | --- | --- | --- | --- |
|  | F | p | F | p | F | p | | F | p | |
|  |  |  |  |  |  |  | |  |  | |
| **Scene construction** | | | | | | | | | | |
| Experiential index | 0.03 | 0.96 | 0.08 | 0.92 | 0.00 | 0.99 | | 0.26 | 0.88 | |
| Spatial references | 0.91 | 0.75 | 0.86 | 0.75 | 0.71 | 0.75 | | 0.36 | 0.88 | |
| Entities present | 0.15 | 0.88 | 0.31 | 0.88 | 0.03 | 0.96 | | 0.73 | 0.75 | |
| Sensory descriptions | 2.51 | 0.52 | 4.19 | 0.42 | 0.84 | 0.75 | | 4.22 | 0.42 | |
| Thoughts/emotions/actions | 1.06 | 0.75 | 0.48 | 0.84 | 1.48 | 0.74 | | 0.05 | 0.96 | |
| Spatial coherence index | 0.40 | 0.88 | 0.14 | 0.88 | 2.33 | 0.56 | | 6.13 | 0.42 | |
|  |  |  |  |  |  |  | |  |  | |
| **Autobiographical interview** | | | | | | | | | | |
| Internal details | 0.14 | 0.95 | 0.10 | 0.95 | 0.14 | 0.95 | | 0.03 | 0.96 | |
| Internal events | 0.82 | 0.95 | 0.89 | 0.95 | 0.56 | 0.95 | | 0.11 | 0.95 | |
| Internal time | 0.00 | 1.00 | 0.33 | 0.95 | 0.31 | 0.95 | | 2.67 | 0.95 | |
| Internal place | 0.97 | 0.95 | 0.51 | 0.95 | 1.23 | 0.95 | | 0.27 | 0.95 | |
| Internal perceptual | 0.03 | 0.96 | 0.40 | 0.95 | 0.09 | 0.95 | | 1.35 | 0.95 | |
| Internal thoughts/emotions | 0.09 | 0.95 | 0.17 | 0.95 | 0.02 | 0.98 | | 0.34 | 0.95 | |
| Vividness ratings | 1.22 | 0.95 | 2.22 | 0.95 | 0.33 | 0.95 | | 1.59 | 0.95 | |
|  |  |  |  |  |  |  | |  |  | |
| **Future thinking** | | | | | | | | | | |
| Experiential index | 0.27 | 0.95 | 0.46 | 0.95 | 0.08 | 0.95 | | 0.39 | 0.95 | |
| Spatial references | 1.59 | 0.83 | 1.10 | 0.86 | 1.66 | 0.83 | | 0.04 | 0.97 | |
| Entities present | 0.68 | 0.95 | 0.63 | 0.95 | 0.54 | 0.95 | | 0.03 | 0.97 | |
| Sensory descriptions | 0.03 | 0.97 | 0.54 | 0.95 | 0.16 | 0.95 | | 2.77 | 0.54 | |
| Thoughts/emotions/actions | 3.11 | 0.54 | 2.95 | 0.54 | 2.43 | 0.58 | | 0.26 | 0.95 | |
| Spatial coherence index | 1.36 | 0.83 | 0.25 | 0.95 | 2.75 | 0.54 | | 1.54 | 0.83 | |
|  |  |  |  |  |  |  | |  |  | |
| **Navigation** | | | | | | | | | | |
| Overall navigation score | 6.28 | 0.37 | 4.75 | 0.40 | 6.08 | 0.37 | | 0.07 | 0.96 | |
| Scene recognition | 0.12 | 0.96 | 0.17 | 0.96 | 0.05 | 0.96 | | 0.06 | 0.96 | |
| Proximity judgements | 0.08 | 0.96 | 0.09 | 0.96 | 0.64 | 0.96 | | 1.75 | 0.96 | |
| Route knowledge | 0.04 | 0.96 | 0.07 | 0.96 | 0.01 | 0.98 | | 0.07 | 0.96 | |
| Sketch map | 5.25 | 0.37 | 2.92 | 0.79 | 6.44 | 0.37 | | 0.26 | 0.96 | |
|  |  |  |  |  |  |  | |  |  | |

P values are Benjamini-Hochberg false discovery rate corrected at p < 0.05.

**Table S20.** Comparison of hippocampal grey matter PD when dividing the sample into two groups determined by their median performance on each cognitive task, with age, gender, total intracranial volume and MRI scanner included as covariates.

| Performance variable | Whole hippocampus | | Anterior hippocampus | | Posterior hippocampus | | Posterior/Anterior hippocampus ratio | | |  |
| --- | --- | --- | --- | --- | --- | --- | --- | --- | --- | --- |
|  | F | p | F | p | F | p | | F | p | |
|  |  |  |  |  |  |  | |  |  | |
| **Scene construction** | | | | | | | | | | |
| Experiential index | 0.76 | 0.75 | 1.00 | 0.75 | 0.25 | 0.88 | | 0.69 | 0.75 | |
| Spatial references | 0.32 | 0.88 | 0.86 | 0.75 | 0.00 | 0.99 | | 1.33 | 0.75 | |
| Entities present | 1.75 | 0.72 | 2.00 | 0.66 | 0.78 | 0.75 | | 1.04 | 0.75 | |
| Sensory descriptions | 0.74 | 0.75 | 0.22 | 0.88 | 1.27 | 0.75 | | 0.14 | 0.88 | |
| Thoughts/emotions/actions | 0.04 | 0.96 | 0.27 | 0.88 | 0.05 | 0.96 | | 0.66 | 0.76 | |
| Spatial coherence index | 0.44 | 0.85 | 0.01 | 0.97 | 1.44 | 0.74 | | 0.79 | 0.75 | |
|  |  |  |  |  |  |  | |  |  | |
| **Autobiographical interview** | | | | | | | | | | |
| Internal details | 0.20 | 0.95 | 1.64 | 0.95 | 0.45 | 0.95 | | 4.70 | 0.95 | |
| Internal events | 0.26 | 0.95 | 0.80 | 0.95 | 0.01 | 0.98 | | 1.45 | 0.95 | |
| Internal time | 1.87 | 0.95 | 1.03 | 0.95 | 2.12 | 0.95 | | 0.00 | 1.00 | |
| Internal place | 0.77 | 0.95 | 0.80 | 0.95 | 0.41 | 0.95 | | 0.33 | 0.95 | |
| Internal perceptual | 0.22 | 0.95 | 0.30 | 0.95 | 0.06 | 0.95 | | 0.23 | 0.95 | |
| Internal thoughts/emotions | 0.10 | 0.95 | 1.08 | 0.95 | 0.43 | 0.95 | | 3.31 | 0.95 | |
| Vividness ratings | 0.49 | 0.95 | 0.06 | 0.95 | 1.18 | 0.95 | | 0.41 | 0.95 | |
|  |  |  |  |  |  |  | |  |  | |
| **Future thinking** | | | | | | | | | | |
| Experiential index | 0.44 | 0.95 | 0.60 | 0.95 | 0.14 | 0.95 | | 0.34 | 0.95 | |
| Spatial references | 1.21 | 0.83 | 1.78 | 0.80 | 0.29 | 0.95 | | 1.41 | 0.83 | |
| Entities present | 0.01 | 0.98 | 0.01 | 0.98 | 0.01 | 0.98 | | 0.00 | 0.99 | |
| Sensory descriptions | 0.02 | 0.98 | 0.06 | 0.95 | 0.00 | 1.00 | | 0.10 | 0.95 | |
| Thoughts/emotions/actions | 2.92 | 0.54 | 1.22 | 0.83 | 4.11 | 0.48 | | 0.13 | 0.95 | |
| Spatial coherence index | 0.28 | 0.95 | 0.26 | 0.95 | 0.18 | 0.95 | | 0.08 | 0.95 | |
|  |  |  |  |  |  |  | |  |  | |
| **Navigation** | | | | | | | | | | |
| Overall navigation score | 0.04 | 0.96 | 0.00 | 1.00 | 0.14 | 0.96 | | 0.09 | 0.96 | |
| Scene recognition | 0.12 | 0.96 | 0.00 | 0.98 | 0.38 | 0.96 | | 0.20 | 0.96 | |
| Proximity judgements | 1.99 | 0.96 | 2.40 | 0.89 | 0.80 | 0.96 | | 1.25 | 0.96 | |
| Route knowledge | 0.21 | 0.96 | 0.04 | 0.96 | 1.34 | 0.96 | | 1.51 | 0.96 | |
| Sketch map | 0.25 | 0.96 | 0.05 | 0.96 | 0.50 | 0.96 | | 0.08 | 0.96 | |
|  |  |  |  |  |  |  | |  |  | |

P values are Benjamini-Hochberg false discovery rate corrected at p < 0.05.

**Table S21.** Comparison of hippocampal grey matter R_1_ when dividing the sample into two groups determined by their median performance on each cognitive task, with age, gender, total intracranial volume and MRI scanner included as covariates.

| Performance variable | Whole hippocampus | | Anterior hippocampus | | Posterior hippocampus | | Posterior/Anterior hippocampus ratio | | |  |
| --- | --- | --- | --- | --- | --- | --- | --- | --- | --- | --- |
|  | F | p | F | p | F | p | | F | p | |
|  |  |  |  |  |  |  | |  |  | |
| **Scene construction** | | | | | | | | | | |
| Experiential index | 2.75 | 0.50 | 3.60 | 0.47 | 1.68 | 0.72 | | 0.92 | 0.75 | |
| Spatial references | 4.13 | 0.42 | 4.83 | 0.42 | 2.88 | 0.50 | | 0.64 | 0.76 | |
| Entities present | 6.71 | 0.42 | 5.72 | 0.42 | 6.27 | 0.42 | | 0.01 | 0.97 | |
| Sensory descriptions | 0.09 | 0.92 | 0.18 | 0.88 | 0.03 | 0.96 | | 0.18 | 0.88 | |
| Thoughts/emotions/actions | 2.83 | 0.50 | 1.63 | 0.72 | 3.49 | 0.47 | | 0.61 | 0.76 | |
| Spatial coherence index | 0.24 | 0.88 | 0.20 | 0.88 | 0.23 | 0.88 | | 0.00 | 0.99 | |
|  |  |  |  |  |  |  | |  |  | |
| **Autobiographical interview** | | | | | | | | | | |
| Internal details | 7.60 | 0.41 | 3.72 | 0.95 | 10.23 | 0.20 | | 2.75 | 0.95 | |
| Internal events | 3.42 | 0.95 | 1.34 | 0.95 | 5.09 | 0.95 | | 2.30 | 0.95 | |
| Internal time | 3.73 | 0.95 | 2.47 | 0.95 | 4.20 | 0.95 | | 0.31 | 0.95 | |
| Internal place | 0.21 | 0.95 | 0.12 | 0.95 | 0.26 | 0.95 | | 0.12 | 0.95 | |
| Internal perceptual | 1.05 | 0.95 | 0.79 | 0.95 | 1.08 | 0.95 | | 0.01 | 0.98 | |
| Internal thoughts/emotions | 0.12 | 0.95 | 0.00 | 1.00 | 0.34 | 0.95 | | 0.63 | 0.95 | |
| Vividness ratings | 0.05 | 0.95 | 0.37 | 0.95 | 0.01 | 0.98 | | 0.92 | 0.95 | |
|  |  |  |  |  |  |  | |  |  | |
| **Future thinking** | | | | | | | | | | |
| Experiential index | 1.04 | 0.87 | 0.87 | 0.94 | 0.99 | 0.88 | | 0.00 | 1.00 | |
| Spatial references | 4.19 | 0.48 | 4.00 | 0.48 | 3.56 | 0.53 | | 0.06 | 0.95 | |
| Entities present | 5.17 | 0.48 | 4.14 | 0.48 | 5.08 | 0.48 | | 0.06 | 0.95 | |
| Sensory descriptions | 0.41 | 0.95 | 0.26 | 0.95 | 0.47 | 0.95 | | 0.06 | 0.95 | |
| Thoughts/emotions/actions | 3.89 | 0.48 | 1.47 | 0.83 | 5.90 | 0.48 | | 2.93 | 0.54 | |
| Spatial coherence index | 0.24 | 0.95 | 0.11 | 0.95 | 0.34 | 0.95 | | 0.08 | 0.95 | |
|  |  |  |  |  |  |  | |  |  | |
| **Navigation** | | | | | | | | | | |
| Overall navigation score | 0.30 | 0.96 | 0.33 | 0.96 | 0.23 | 0.96 | | 0.03 | 0.96 | |
| Scene recognition | 0.01 | 0.98 | 0.00 | 0.98 | 0.05 | 0.96 | | 0.19 | 0.96 | |
| Proximity judgements | 1.42 | 0.96 | 1.42 | 0.96 | 1.16 | 0.96 | | 0.08 | 0.96 | |
| Route knowledge | 0.06 | 0.96 | 0.31 | 0.96 | 0.83 | 0.96 | | 5.45 | 0.37 | |
| Sketch map | 0.11 | 0.96 | 0.10 | 0.96 | 0.10 | 0.96 | | 0.00 | 1.00 | |
|  |  |  |  |  |  |  | |  |  | |

P values are Benjamini-Hochberg false discovery rate corrected at p < 0.05.

**Table S22.** Comparison of hippocampal grey matter R_2_* when dividing the sample into two groups determined by their median performance on each cognitive task, with age, gender, total intracranial volume and MRI scanner included as covariates.

| Performance variable | Whole hippocampus | | Anterior hippocampus | | Posterior hippocampus | | Posterior/Anterior hippocampus ratio | | |  |
| --- | --- | --- | --- | --- | --- | --- | --- | --- | --- | --- |
|  | F | p | F | p | F | p | | F | p | |
|  |  |  |  |  |  |  | |  |  | |
| **Scene construction** | | | | | | | | | | |
| Experiential index | 1.72 | 0.72 | 1.52 | 0.74 | 1.18 | 0.75 | | 0.22 | 0.88 | |
| Spatial references | 4.36 | 0.42 | 2.76 | 0.50 | 4.13 | 0.42 | | 0.00 | 0.99 | |
| Entities present | 3.06 | 0.50 | 1.38 | 0.75 | 3.71 | 0.47 | | 0.36 | 0.88 | |
| Sensory descriptions | 0.78 | 0.75 | 1.25 | 0.75 | 0.20 | 0.88 | | 0.23 | 0.88 | |
| Thoughts/emotions/actions | 0.92 | 0.75 | 0.01 | 0.97 | 3.37 | 0.47 | | 2.64 | 0.51 | |
| Spatial coherence index | 0.02 | 0.96 | 0.12 | 0.89 | 0.01 | 0.97 | | 0.09 | 0.92 | |
|  |  |  |  |  |  |  | |  |  | |
| **Autobiographical interview** | | | | | | | | | | |
| Internal details | 1.07 | 0.95 | 0.17 | 0.95 | 2.08 | 0.95 | | 0.38 | 0.95 | |
| Internal events | 0.01 | 0.98 | 0.44 | 0.95 | 0.75 | 0.95 | | 2.22 | 0.95 | |
| Internal time | 0.08 | 0.95 | 0.74 | 0.95 | 0.14 | 0.95 | | 1.49 | 0.95 | |
| Internal place | 0.91 | 0.95 | 0.55 | 0.95 | 0.91 | 0.95 | | 0.08 | 0.95 | |
| Internal perceptual | 1.16 | 0.95 | 0.37 | 0.95 | 1.71 | 0.95 | | 0.30 | 0.95 | |
| Internal thoughts/emotions | 0.07 | 0.95 | 0.07 | 0.95 | 0.04 | 0.96 | | 0.05 | 0.95 | |
| Vividness ratings | 0.23 | 0.95 | 0.70 | 0.95 | 0.00 | 1.00 | | 0.87 | 0.95 | |
|  |  |  |  |  |  |  | |  |  | |
| **Future thinking** | | | | | | | | | | |
| Experiential index | 0.04 | 0.97 | 0.21 | 0.95 | 0.01 | 0.98 | | 0.17 | 0.95 | |
| Spatial references | 2.63 | 0.54 | 4.06 | 0.48 | 0.72 | 0.95 | | 1.33 | 0.83 | |
| Entities present | 2.67 | 0.54 | 4.27 | 0.48 | 0.67 | 0.95 | | 1.97 | 0.74 | |
| Sensory descriptions | 0.08 | 0.95 | 0.24 | 0.95 | 0.00 | 1.00 | | 0.07 | 0.95 | |
| Thoughts/emotions/actions | 0.44 | 0.95 | 0.01 | 0.98 | 1.20 | 0.83 | | 0.48 | 0.95 | |
| Spatial coherence index | 0.57 | 0.95 | 0.75 | 0.95 | 0.22 | 0.95 | | 0.10 | 0.95 | |
|  |  |  |  |  |  |  | |  |  | |
| **Navigation** | | | | | | | | | | |
| Overall navigation score | 1.25 | 0.96 | 0.16 | 0.96 | 2.53 | 0.89 | | 1.40 | 0.96 | |
| Scene recognition | 0.82 | 0.96 | 0.14 | 0.96 | 1.53 | 0.96 | | 0.73 | 0.96 | |
| Proximity judgements | 0.08 | 0.96 | 0.01 | 0.98 | 0.16 | 0.96 | | 0.02 | 0.98 | |
| Route knowledge | 0.11 | 0.96 | 0.01 | 0.98 | 0.44 | 0.96 | | 0.71 | 0.96 | |
| Sketch map | 1.38 | 0.96 | 0.06 | 0.96 | 3.50 | 0.72 | | 3.11 | 0.79 | |
|  |  |  |  |  |  |  | |  |  | |

P values are Benjamini-Hochberg false discovery rate corrected at p < 0.05.

**Table S23.** Partial correlations between task performance and hippocampal grey matter MT saturation in the low performing participants only (as determined by a median split for each task) with age, gender, total intracranial volume and MRI scanner as covariates.

| Performance variable | Whole hippocampus | | Anterior hippocampus | | Posterior hippocampus | | Posterior/Anterior hippocampus ratio | | |  |
| --- | --- | --- | --- | --- | --- | --- | --- | --- | --- | --- |
|  | r | p | r | p | r | p | | r | p | |
|  |  |  |  |  |  |  | |  |  | |
| **Scene construction** | | | | | | | | | | |
| Experiential index | -0.17 | 0.98 | -0.15 | 0.98 | -0.16 | 0.98 | | 0.008 | 0.99 | |
| Spatial references | -0.22 | 0.59 | -0.15 | 0.98 | -0.24 | 0.52 | | -0.12 | 0.98 | |
| Entities present | -0.010 | 0.99 | -0.009 | 0.99 | -0.010 | 0.99 | | -0.019 | 0.99 | |
| Sensory descriptions | -0.12 | 0.98 | -0.033 | 0.98 | -0.19 | 0.96 | | -0.18 | 0.96 | |
| Thoughts/emotions/actions | -0.17 | 0.98 | -0.16 | 0.98 | -0.14 | 0.98 | | 0.10 | 0.98 | |
| Spatial coherence index | -0.003 | 0.99 | -0.044 | 0.98 | 0.038 | 0.98 | | 0.092 | 0.98 | |
|  |  |  |  |  |  |  | |  |  | |
| **Autobiographical interview** | | | | | | | | | | |
| Internal details | -0.10 | 0.78 | -0.036 | 0.91 | -0.16 | 0.78 | | -0.099 | 0.78 | |
| Internal events | 0.14 | 0.78 | 0.15 | 0.78 | 0.11 | 0.78 | | -0.063 | 0.79 | |
| Internal time | 0.035 | 0.90 | 0.060 | 0.79 | 0.005 | 1.00 | | -0.088 | 0.78 | |
| Internal place | 0.010 | 0.98 | 0.053 | 0.79 | -0.036 | 0.90 | | -0.13 | 0.78 | |
| Internal perceptual | -0.009 | 0.99 | 0.086 | 0.78 | -0.11 | 0.78 | | -0.21 | 0.72 | |
| Internal thoughts/emotions | -0.067 | 0.79 | -0.012 | 0.98 | -0.12 | 0.78 | | -0.088 | 0.78 | |
| Vividness ratings | -0.084 | 0.78 | -0.004 | 1.00 | -0.16 | 0.78 | | -0.16 | 0.78 | |
|  |  |  |  |  |  |  | |  |  | |
| **Future thinking** | | | | | | | | | | |
| Experiential index | -0.095 | 0.79 | -0.036 | 0.93 | -0.14 | 0.78 | | -0.099 | 0.79 | |
| Spatial references | -0.087 | 0.79 | -0.032 | 0.93 | -0.13 | 0.78 | | -0.11 | 0.78 | |
| Entities present | -0.029 | 0.93 | 0.020 | 0.93 | -0.070 | 0.79 | | -0.104 | 0.78 | |
| Sensory descriptions | -0.16 | 0.78 | -0.13 | 0.78 | -0.18 | 0.78 | | -0.018 | 0.93 | |
| Thoughts/emotions/actions | -0.012 | 0.95 | -0.013 | 0.95 | -0.009 | 0.95 | | -0.008 | 0.95 | |
| Spatial coherence index | -0.020 | 0.93 | -0.031 | 0.93 | -0.008 | 0.95 | | 0.020 | 0.93 | |
|  |  |  |  |  |  |  | |  |  | |
| **Navigation** | | | | | | | | | | |
| Overall navigation score | 0.022 | 0.94 | 0.13 | 0.60 | -0.083 | 0.71 | | -0.27 | 0.46 | |
| Scene recognition | 0.13 | 0.60 | 0.13 | 0.60 | 0.11 | 0.60 | | -0.054 | 0.74 | |
| Proximity judgements | -0.088 | 0.60 | -0.11 | 0.60 | -0.056 | 0.74 | | 0.092 | 0.60 | |
| Route knowledge | 0.059 | 0.75 | 0.10 | 0.60 | 0.013 | 0.94 | | -0.13 | 0.60 | |
| Sketch map | -0.071 | 0.74 | 0.016 | 0.94 | -0.15 | 0.60 | | -0.18 | 0.60 | |
|  |  |  |  |  |  |  | |  |  | |

P values are Benjamini-Hochberg false discovery rate corrected at p < 0.05.

**Table S24.** Partial correlations between task performance and hippocampal grey matter PD in the low performing participants only (as determined by a median split for each task) with age, gender, total intracranial volume and MRI scanner as covariates.

| Performance variable | Whole hippocampus | | Anterior hippocampus | | Posterior hippocampus | | Posterior/Anterior hippocampus ratio | | |  |
| --- | --- | --- | --- | --- | --- | --- | --- | --- | --- | --- |
|  | r | p | r | p | r | p | | r | p | |
|  |  |  |  |  |  |  | |  |  | |
| **Scene construction** | | | | | | | | | | |
| Experiential index | 0.052 | 0.98 | 0.047 | 0.98 | 0.045 | 0.98 | | -0.016 | 0.99 | |
| Spatial references | 0.002 | 0.99 | -0.024 | 0.99 | 0.033 | 0.98 | | 0.052 | 0.98 | |
| Entities present | 0.072 | 0.98 | 0.079 | 0.98 | 0.044 | 0.98 | | -0.054 | 0.98 | |
| Sensory descriptions | 0.044 | 0.98 | 0.055 | 0.98 | 0.021 | 0.99 | | -0.052 | 0.98 | |
| Thoughts/emotions/actions | -0.063 | 0.98 | -0.068 | 0.98 | -0.036 | 0.98 | | 0.046 | 0.98 | |
| Spatial coherence index | 0.069 | 0.98 | 0.11 | 0.98 | 0.002 | 0.99 | | -0.13 | 0.98 | |
|  |  |  |  |  |  |  | |  |  | |
| **Autobiographical interview** | | | | | | | | | | |
| Internal details | -0.076 | 0.79 | -0.094 | 0.78 | -0.029 | 0.94 | | 0.086 | 0.78 | |
| Internal events | -0.092 | 0.78 | -0.057 | 0.79 | -0.11 | 0.78 | | -0.013 | 0.98 | |
| Internal time | -0.059 | 0.79 | -0.088 | 0.78 | 0.00 | 1.00 | | 0.092 | 0.78 | |
| Internal place | -0.12 | 0.78 | -0.10 | 0.78 | -0.13 | 0.78 | | 0.033 | 0.91 | |
| Internal perceptual | -0.13 | 0.78 | -0.13 | 0.78 | -0.086 | 0.78 | | 0.089 | 0.78 | |
| Internal thoughts/emotions | 0.12 | 0.78 | 0.089 | 0.78 | 0.12 | 0.78 | | -0.006 | 1.00 | |
| Vividness ratings | -0.20 | 0.77 | -0.15 | 0.78 | -0.22 | 0.72 | | -0.002 | 1.00 | |
|  |  |  |  |  |  |  | |  |  | |
| **Future thinking** | | | | | | | | | | |
| Experiential index | 0.073 | 0.79 | 0.12 | 0.78 | 0.00 | 1.00 | | -0.15 | 0.78 | |
| Spatial references | 0.11 | 0.78 | 0.098 | 0.78 | 0.098 | 0.78 | | -0.040 | 0.91 | |
| Entities present | 0.041 | 0.91 | 0.041 | 0.91 | 0.030 | 0.93 | | -0.026 | 0.93 | |
| Sensory descriptions | 0.10 | 0.78 | 0.10 | 0.78 | 0.071 | 0.79 | | -0.064 | 0.82 | |
| Thoughts/emotions/actions | 0.085 | 0.79 | 0.12 | 0.78 | 0.008 | 0.95 | | -0.13 | 0.78 | |
| Spatial coherence index | 0.083 | 0.79 | 0.12 | 0.78 | 0.022 | 0.93 | | -0.12 | 0.78 | |
|  |  |  |  |  |  |  | |  |  | |
| **Navigation** | | | | | | | | | | |
| Overall navigation score | -0.066 | 0.74 | -0.11 | 0.60 | 0.002 | 0.99 | | 0.12 | 0.60 | |
| Scene recognition | -0.15 | 0.60 | -0.13 | 0.60 | -0.12 | 0.60 | | 0.055 | 0.74 | |
| Proximity judgements | 0.085 | 0.60 | 0.023 | 0.93 | 0.13 | 0.60 | | 0.085 | 0.60 | |
| Route knowledge | -0.12 | 0.60 | -0.098 | 0.60 | -0.11 | 0.60 | | 0.017 | 0.94 | |
| Sketch map | 0.003 | 0.98 | -0.053 | 0.78 | 0.062 | 0.74 | | 0.11 | 0.60 | |
|  |  |  |  |  |  |  | |  |  | |

P values are Benjamini-Hochberg false discovery rate corrected at p < 0.05.

**Table S25.** Partial correlations between task performance and hippocampal grey matter R_1_ in the low performing participants only (as determined by a median split for each task) with age, gender, total intracranial volume and MRI scanner as covariates.

| Performance variable | Whole hippocampus | | Anterior hippocampus | | Posterior hippocampus | | Posterior/Anterior hippocampus ratio | | |  |
| --- | --- | --- | --- | --- | --- | --- | --- | --- | --- | --- |
|  | r | p | r | p | r | p | | r | p | |
|  |  |  |  |  |  |  | |  |  | |
| **Scene construction** | | | | | | | | | | |
| Experiential index | 0.007 | 0.99 | 0.036 | 0.98 | -0.018 | 0.99 | | -0.076 | 0.98 | |
| Spatial references | 0.013 | 0.99 | 0.039 | 0.98 | -0.008 | 0.99 | | -0.073 | 0.98 | |
| Entities present | -0.006 | 0.99 | 0.025 | 0.99 | -0.030 | 0.99 | | -0.075 | 0.98 | |
| Sensory descriptions | -0.042 | 0.98 | -0.040 | 0.98 | -0.042 | 0.98 | | -0.001 | 0.99 | |
| Thoughts/emotions/actions | 0.061 | 0.98 | 0.058 | 0.98 | 0.052 | 0.98 | | -0.004 | 0.99 | |
| Spatial coherence index | -0.080 | 0.98 | -0.11 | 0.98 | -0.053 | 0.98 | | 0.093 | 0.98 | |
|  |  |  |  |  |  |  | |  |  | |
| **Autobiographical interview** | | | | | | | | | | |
| Internal details | 0.087 | 0.78 | 0.10 | 0.78 | 0.061 | 0.79 | | -0.069 | 0.79 | |
| Internal events | 0.277 | 0.35 | 0.25 | 0.43 | 0.27 | 0.35 | | 0.005 | 1.00 | |
| Internal time | 0.066 | 0.79 | 0.090 | 0.78 | 0.032 | 0.91 | | -0.085 | 0.78 | |
| Internal place | -0.011 | 0.98 | -0.022 | 0.97 | 0.00 | 1.00 | | 0.039 | 0.89 | |
| Internal perceptual | 0.14 | 0.78 | 0.15 | 0.78 | 0.12 | 0.78 | | -0.065 | 0.79 | |
| Internal thoughts/emotions | 0.021 | 0.98 | 0.029 | 0.94 | 0.010 | 0.98 | | -0.018 | 0.98 | |
| Vividness ratings | 0.042 | 0.89 | 0.054 | 0.79 | 0.027 | 0.94 | | -0.054 | 0.79 | |
|  |  |  |  |  |  |  | |  |  | |
| **Future thinking** | | | | | | | | | | |
| Experiential index | 0.17 | 0.78 | 0.14 | 0.78 | 0.18 | 0.78 | | 0.082 | 0.79 | |
| Spatial references | 0.13 | 0.78 | 0.10 | 0.78 | 0.14 | 0.78 | | 0.075 | 0.79 | |
| Entities present | 0.050 | 0.89 | 0.055 | 0.88 | 0.042 | 0.91 | | -0.028 | 0.93 | |
| Sensory descriptions | 0.062 | 0.82 | 0.070 | 0.79 | 0.050 | 0.89 | | -0.041 | 0.91 | |
| Thoughts/emotions/actions | 0.16 | 0.78 | 0.11 | 0.78 | 0.17 | 0.78 | | 0.059 | 0.85 | |
| Spatial coherence index | -0.13 | 0.78 | -0.15 | 0.78 | -0.11 | 0.78 | | 0.069 | 0.79 | |
|  |  |  |  |  |  |  | |  |  | |
| **Navigation** | | | | | | | | | | |
| Overall navigation score | -0.038 | 0.87 | 0.016 | 0.94 | -0.079 | 0.72 | | -0.13 | 0.60 | |
| Scene recognition | 0.049 | 0.77 | 0.059 | 0.74 | 0.036 | 0.84 | | -0.042 | 0.79 | |
| Proximity judgements | 0.010 | 0.94 | 0.043 | 0.78 | -0.018 | 0.94 | | -0.091 | 0.60 | |
| Route knowledge | 0.12 | 0.60 | 0.093 | 0.63 | 0.14 | 0.60 | | 0.077 | 0.72 | |
| Sketch map | -0.065 | 0.74 | -0.013 | 0.94 | -0.10 | 0.60 | | -0.12 | 0.60 | |
|  |  |  |  |  |  |  | |  |  | |

P values are Benjamini-Hochberg false discovery rate corrected at p < 0.05.

**Table S26.** Partial correlations between task performance and hippocampal grey matter R_2_* in the low performing participants only (as determined by a median split for each task) with age, gender, total intracranial volume and MRI scanner as covariates.

| Performance variable | Whole hippocampus | | Anterior hippocampus | | Posterior hippocampus | | Posterior/Anterior hippocampus ratio | | |  |
| --- | --- | --- | --- | --- | --- | --- | --- | --- | --- | --- |
|  | r | p | r | p | r | p | | r | p | |
|  |  |  |  |  |  |  | |  |  | |
| **Scene construction** | | | | | | | | | | |
| Experiential index | 0.063 | 0.98 | 0.053 | 0.98 | 0.059 | 0.98 | | -0.014 | 0.99 | |
| Spatial references | 0.23 | 0.52 | 0.14 | 0.98 | 0.24 | 0.52 | | 0.047 | 0.98 | |
| Entities present | 0.11 | 0.98 | 0.11 | 0.98 | 0.079 | 0.98 | | -0.045 | 0.98 | |
| Sensory descriptions | 0.006 | 0.99 | -0.012 | 0.99 | 0.018 | 0.99 | | 0.037 | 0.98 | |
| Thoughts/emotions/actions | 0.13 | 0.98 | 0.11 | 0.98 | 0.11 | 0.98 | | -0.041 | 0.98 | |
| Spatial coherence index | -0.10 | 0.98 | -0.074 | 0.98 | -0.11 | 0.98 | | -0.033 | 0.98 | |
|  |  |  |  |  |  |  | |  |  | |
| **Autobiographical interview** | | | | | | | | | | |
| Internal details | 0.080 | 0.79 | 0.071 | 0.79 | 0.075 | 0.79 | | -0.014 | 0.98 | |
| Internal events | 0.18 | 0.78 | 0.19 | 0.78 | 0.13 | 0.78 | | -0.12 | 0.78 | |
| Internal time | -0.012 | 0.98 | -0.067 | 0.79 | 0.058 | 0.79 | | 0.15 | 0.78 | |
| Internal place | 0.082 | 0.78 | 0.057 | 0.79 | 0.091 | 0.78 | | 0.041 | 0.89 | |
| Internal perceptual | 0.17 | 0.78 | 0.19 | 0.78 | 0.10 | 0.78 | | -0.10 | 0.78 | |
| Internal thoughts/emotions | 0.091 | 0.78 | 0.084 | 0.78 | 0.080 | 0.79 | | -0.021 | 0.98 | |
| Vividness ratings | 0.016 | 0.98 | -0.015 | 0.98 | 0.047 | 0.86 | | 0.097 | 0.78 | |
|  |  |  |  |  |  |  | |  |  | |
| **Future thinking** | | | | | | | | | | |
| Experiential index | 0.12 | 0.78 | 0.10 | 0.78 | 0.12 | 0.78 | | 0.012 | 0.95 | |
| Spatial references | 0.16 | 0.78 | 0.11 | 0.78 | 0.16 | 0.78 | | 0.076 | 0.79 | |
| Entities present | 0.046 | 0.91 | 0.052 | 0.89 | 0.033 | 0.93 | | -0.008 | 0.95 | |
| Sensory descriptions | 0.070 | 0.79 | 0.027 | 0.93 | 0.086 | 0.79 | | 0.069 | 0.79 | |
| Thoughts/emotions/actions | -0.082 | 0.79 | -0.062 | 0.82 | -0.089 | 0.79 | | -0.020 | 0.93 | |
| Spatial coherence index | -0.084 | 0.79 | -0.080 | 0.79 | -0.073 | 0.79 | | 0.021 | 0.93 | |
|  |  |  |  |  |  |  | |  |  | |
| **Navigation** | | | | | | | | | | |
| Overall navigation score | 0.16 | 0.60 | 0.12 | 0.60 | 0.16 | 0.60 | | 0.019 | 0.94 | |
| Scene recognition | 0.12 | 0.60 | 0.094 | 0.60 | 0.11 | 0.60 | | 0.008 | 0.95 | |
| Proximity judgements | 0.072 | 0.67 | 0.075 | 0.66 | 0.057 | 0.74 | | -0.014 | 0.94 | |
| Route knowledge | 0.10 | 0.60 | 0.067 | 0.74 | 0.11 | 0.60 | | 0.034 | 0.88 | |
| Sketch map | 0.14 | 0.60 | 0.14 | 0.60 | 0.11 | 0.60 | | -0.049 | 0.79 | |
|  |  |  |  |  |  |  | |  |  | |

P values are Benjamini-Hochberg false discovery rate corrected at p < 0.05.

**Table S27.** Partial correlations between performance and hippocampal grey matter MT saturation in the high performing participants only (as determined by a median split for each task) with age, gender, total intracranial volume and MRI scanner as covariates.

| Performance variable | Whole hippocampus | | Anterior hippocampus | | Posterior hippocampus | | Posterior/Anterior hippocampus ratio | | |  |
| --- | --- | --- | --- | --- | --- | --- | --- | --- | --- | --- |
|  | r | p | r | p | r | p | | r | p | |
|  |  |  |  |  |  |  | |  |  | |
| **Scene construction** | | | | | | | | | | |
| Experiential index | -0.13 | 0.77 | -0.14 | 0.77 | -0.10 | 0.77 | | 0.090 | 0.77 | |
| Spatial references | 0.001 | 1.00 | -0.045 | 0.91 | 0.051 | 0.91 | | 0.11 | 0.77 | |
| Entities present | -0.12 | 0.77 | -0.13 | 0.77 | -0.099 | 0.77 | | 0.099 | 0.77 | |
| Sensory descriptions | -0.036 | 0.96 | -0.095 | 0.77 | 0.027 | 0.99 | | 0.17 | 0.77 | |
| Thoughts/emotions/actions | -0.18 | 0.77 | -0.22 | 0.77 | -0.12 | 0.77 | | 0.19 | 0.77 | |
| Spatial coherence index | -0.13 | 0.77 | -0.14 | 0.77 | -0.10 | 0.77 | | 0.11 | 0.77 | |
|  |  |  |  |  |  |  | |  |  | |
| **Autobiographical interview** | | | | | | | | | | |
| Internal details | 0.009 | 0.99 | -0.013 | 0.99 | 0.027 | 0.95 | | 0.047 | 0.93 | |
| Internal events | 0.086 | 0.81 | 0.088 | 0.81 | 0.074 | 0.81 | | -0.043 | 0.93 | |
| Internal time | 0.14 | 0.75 | 0.16 | 0.75 | 0.10 | 0.81 | | -0.091 | 0.81 | |
| Internal place | -0.073 | 0.81 | -0.10 | 0.81 | -0.040 | 0.94 | | 0.078 | 0.81 | |
| Internal perceptual | 0.016 | 0.99 | 0.011 | 0.99 | 0.019 | 0.99 | | 0.008 | 0.99 | |
| Internal thoughts/emotions | -0.032 | 0.94 | -0.062 | 0.82 | -0.001 | 1.00 | | 0.096 | 0.81 | |
| Vividness ratings | -0.043 | 0.93 | -0.076 | 0.81 | -0.011 | 0.99 | | 0.087 | 0.81 | |
|  |  |  |  |  |  |  | |  |  | |
| **Future thinking** | | | | | | | | | | |
| Experiential index | -0.28 | 0.17 | -0.32 | 0.11 | -0.20 | 0.36 | | 0.25 | 0.28 | |
| Spatial references | -0.17 | 0.49 | -0.20 | 0.39 | -0.122 | 0.74 | | 0.15 | 0.59 | |
| Entities present | -0.22 | 0.34 | -0.23 | 0.32 | -0.19 | 0.39 | | 0.15 | 0.54 | |
| Sensory descriptions | -0.10 | 0.79 | -0.089 | 0.80 | -0.097 | 0.79 | | 0.061 | 0.84 | |
| Thoughts/emotions/actions | 0.21 | 0.35 | 0.16 | 0.49 | 0.24 | 0.29 | | 0.053 | 0.84 | |
| Spatial coherence index | -0.12 | 0.74 | -0.098 | 0.79 | -0.12 | 0.74 | | 0.031 | 0.88 | |
|  |  |  |  |  |  |  | |  |  | |
| **Navigation** | | | | | | | | | | |
| Overall navigation score | 0.077 | 0.99 | 0.059 | 0.99 | 0.084 | 0.99 | | 0.000 | 1.00 | |
| Scene recognition | 0.22 | 0.99 | 0.24 | 0.99 | 0.17 | 0.99 | | -0.14 | 0.99 | |
| Proximity judgements | -0.090 | 0.99 | -0.055 | 0.99 | -0.12 | 0.99 | | -0.047 | 0.99 | |
| Route knowledge | -0.10 | 0.99 | -0.11 | 0.99 | -0.069 | 0.99 | | 0.065 | 0.99 | |
| Sketch map | 0.062 | 0.99 | 0.017 | 1.00 | 0.097 | 0.99 | | 0.074 | 0.99 | |
|  |  |  |  |  |  |  | |  |  | |

P values are Benjamini-Hochberg false discovery rate corrected at p < 0.05.

**Table S28.** Partial correlations between performance and hippocampal grey matter PD in the high performing participants only (as determined by a median split for each task) with age, gender, total intracranial volume and MRI scanner as covariates.

| Performance variable | Whole hippocampus | | Anterior hippocampus | | Posterior hippocampus | | Posterior/Anterior hippocampus ratio | | |  |
| --- | --- | --- | --- | --- | --- | --- | --- | --- | --- | --- |
|  | r | p | r | p | r | p | | r | p | |
|  |  |  |  |  |  |  | |  |  | |
| **Scene construction** | | | | | | | | | | |
| Experiential index | 0.005 | 1.00 | -0.010 | 0.99 | 0.024 | 0.99 | | 0.024 | 0.99 | |
| Spatial references | 0.017 | 0.99 | -0.048 | 0.91 | 0.092 | 0.77 | | 0.14 | 0.77 | |
| Entities present | -0.074 | 0.77 | -0.075 | 0.77 | -0.056 | 0.88 | | 0.045 | 0.91 | |
| Sensory descriptions | -0.11 | 0.77 | -0.12 | 0.77 | -0.070 | 0.79 | | 0.083 | 0.77 | |
| Thoughts/emotions/actions | -0.075 | 0.77 | -0.11 | 0.77 | -0.015 | 0.99 | | 0.14 | 0.77 | |
| Spatial coherence index | -0.094 | 0.77 | -0.12 | 0.77 | -0.042 | 0.91 | | 0.11 | 0.77 | |
|  |  |  |  |  |  |  | |  |  | |
| **Autobiographical interview** | | | | | | | | | | |
| Internal details | 0.12 | 0.81 | 0.15 | 0.75 | 0.062 | 0.82 | | -0.13 | 0.75 | |
| Internal events | 0.10 | 0.81 | 0.17 | 0.65 | 0.003 | 1.00 | | -0.24 | 0.55 | |
| Internal time | 0.017 | 0.99 | -0.011 | 0.99 | 0.046 | 0.93 | | 0.065 | 0.83 | |
| Internal place | -0.034 | 0.94 | -0.048 | 0.93 | -0.011 | 0.99 | | 0.038 | 0.94 | |
| Internal perceptual | 0.072 | 0.81 | 0.13 | 0.75 | -0.011 | 0.99 | | -0.16 | 0.74 | |
| Internal thoughts/emotions | 0.085 | 0.81 | 0.14 | 0.75 | 0.00 | 1.00 | | -0.18 | 0.65 | |
| Vividness ratings | 0.052 | 0.90 | 0.075 | 0.81 | 0.013 | 0.99 | | -0.087 | 0.81 | |
|  |  |  |  |  |  |  | |  |  | |
| **Future thinking** | | | | | | | | | | |
| Experiential index | -0.11 | 0.74 | -0.11 | 0.74 | -0.082 | 0.81 | | 0.053 | 0.84 | |
| Spatial references | -0.044 | 0.88 | -0.071 | 0.83 | 0.004 | 0.98 | | 0.083 | 0.81 | |
| Entities present | -0.061 | 0.84 | -0.080 | 0.81 | -0.023 | 0.90 | | 0.076 | 0.81 | |
| Sensory descriptions | -0.039 | 0.88 | -0.087 | 0.80 | 0.030 | 0.88 | | 0.14 | 0.65 | |
| Thoughts/emotions/actions | 0.069 | 0.83 | 0.071 | 0.83 | 0.055 | 0.84 | | -0.043 | 0.88 | |
| Spatial coherence index | -0.057 | 0.84 | -0.026 | 0.88 | -0.080 | 0.81 | | -0.041 | 0.88 | |
|  |  |  |  |  |  |  | |  |  | |
| **Navigation** | | | | | | | | | | |
| Overall navigation score | 0.033 | 0.99 | 0.009 | 1.00 | 0.058 | 0.99 | | 0.038 | 0.99 | |
| Scene recognition | 0.056 | 0.99 | 0.067 | 0.99 | 0.027 | 1.00 | | -0.065 | 0.99 | |
| Proximity judgements | 0.022 | 1.00 | 0.036 | 1.00 | -0.001 | 1.00 | | -0.049 | 0.99 | |
| Route knowledge | -0.041 | 0.99 | -0.042 | 0.99 | -0.030 | 1.00 | | 0.023 | 1.00 | |
| Sketch map | 0.049 | 0.99 | 0.035 | 0.99 | 0.058 | 0.99 | | 0.002 | 1.00 | |
|  |  |  |  |  |  |  | |  |  | |

P values are Benjamini-Hochberg false discovery rate corrected at p < 0.05.

**Table S29.** Partial correlations between performance and hippocampal grey matter R_1_ in the high performing participants only (as determined by a median split for each task) with age, gender, total intracranial volume and MRI scanner as covariates.

| Performance variable | Whole hippocampus | | Anterior hippocampus | | Posterior hippocampus | | Posterior/Anterior hippocampus ratio | | |  |
| --- | --- | --- | --- | --- | --- | --- | --- | --- | --- | --- |
|  | r | p | r | p | r | p | | r | p | |
|  |  |  |  |  |  |  | |  |  | |
| **Scene construction** | | | | | | | | | | |
| Experiential index | -0.008 | 0.99 | -0.004 | 1.00 | -0.010 | 0.99 | | -0.019 | 0.99 | |
| Spatial references | -0.007 | 0.99 | 0.000 | 1.00 | -0.013 | 0.99 | | -0.019 | 0.99 | |
| Entities present | -0.055 | 0.88 | -0.020 | 0.99 | -0.080 | 0.77 | | -0.10 | 0.77 | |
| Sensory descriptions | 0.17 | 0.77 | 0.15 | 0.77 | 0.17 | 0.77 | | 0.008 | 0.99 | |
| Thoughts/emotions/actions | 0.13 | 0.77 | 0.15 | 0.77 | 0.11 | 0.77 | | -0.095 | 0.77 | |
| Spatial coherence index | 0.075 | 0.77 | 0.083 | 0.77 | 0.058 | 0.88 | | -0.048 | 0.91 | |
|  |  |  |  |  |  |  | |  |  | |
| **Autobiographical interview** | | | | | | | | | | |
| Internal details | -0.084 | 0.81 | -0.082 | 0.81 | -0.080 | 0.81 | | 0.006 | 1.00 | |
| Internal events | -0.056 | 0.86 | -0.081 | 0.81 | -0.031 | 0.94 | | 0.098 | 0.81 | |
| Internal time | -0.12 | 0.81 | -0.073 | 0.82 | -0.15 | 0.75 | | -0.16 | 0.75 | |
| Internal place | 0.15 | 0.75 | 0.11 | 0.81 | 0.16 | 0.75 | | 0.077 | 0.81 | |
| Internal perceptual | 0.090 | 0.81 | 0.068 | 0.81 | 0.097 | 0.81 | | 0.038 | 0.94 | |
| Internal thoughts/emotions | -0.029 | 0.94 | -0.064 | 0.82 | 0.001 | 1.00 | | 0.13 | 0.75 | |
| Vividness ratings | -0.21 | 0.65 | -0.18 | 0.65 | -0.20 | 0.65 | | -0.072 | 0.81 | |
|  |  |  |  |  |  |  | |  |  | |
| **Future thinking** | | | | | | | | | | |
| Experiential index | 0.042 | 0.88 | 0.047 | 0.88 | 0.033 | 0.88 | | -0.026 | 0.88 | |
| Spatial references | 0.12 | 0.74 | 0.12 | 0.74 | 0.094 | 0.80 | | -0.037 | 0.88 | |
| Entities present | 0.066 | 0.84 | 0.096 | 0.79 | 0.032 | 0.88 | | -0.088 | 0.80 | |
| Sensory descriptions | 0.17 | 0.49 | 0.18 | 0.47 | 0.14 | 0.61 | | -0.057 | 0.84 | |
| Thoughts/emotions/actions | -0.065 | 0.84 | -0.072 | 0.83 | -0.055 | 0.84 | | 0.034 | 0.88 | |
| Spatial coherence index | 0.000 | 1.00 | -0.005 | 0.98 | 0.004 | 0.98 | | 0.014 | 0.95 | |
|  |  |  |  |  |  |  | |  |  | |
| **Navigation** | | | | | | | | | | |
| Overall navigation score | -0.11 | 0.99 | -0.10 | 0.99 | -0.11 | 0.99 | | -0.013 | 1.00 | |
| Scene recognition | -0.13 | 0.99 | -0.074 | 0.99 | -0.15 | 0.99 | | -0.11 | 0.99 | |
| Proximity judgements | -0.15 | 0.99 | -0.13 | 0.99 | -0.15 | 0.99 | | 0.001 | 1.00 | |
| Route knowledge | -0.054 | 0.99 | -0.064 | 0.99 | -0.039 | 0.99 | | 0.023 | 1.00 | |
| Sketch map | -0.059 | 0.99 | -0.058 | 0.99 | -0.056 | 0.99 | | 0.000 | 1.00 | |
|  |  |  |  |  |  |  | |  |  | |

P values are Benjamini-Hochberg false discovery rate corrected at p < 0.05.

**Table S30.** Partial correlations between performance and hippocampal grey matter R_2_* in the high performing participants only (as determined by a median split for each task) with age, gender, total intracranial volume and MRI scanner as covariates.

| Performance variable | Whole hippocampus | | Anterior hippocampus | | Posterior hippocampus | | Posterior/Anterior hippocampus ratio | | |  |
| --- | --- | --- | --- | --- | --- | --- | --- | --- | --- | --- |
|  | r | p | r | p | r | p | | r | p | |
|  |  |  |  |  |  |  | |  |  | |
| **Scene construction** | | | | | | | | | | |
| Experiential index | -0.057 | 0.88 | -0.11 | 0.77 | 0.008 | 0.99 | | 0.13 | 0.77 | |
| Spatial references | -0.080 | 0.77 | -0.083 | 0.77 | -0.062 | 0.86 | | 0.021 | 0.99 | |
| Entities present | -0.018 | 0.99 | -0.074 | 0.77 | 0.044 | 0.91 | | 0.15 | 0.77 | |
| Sensory descriptions | -0.002 | 1.00 | -0.077 | 0.77 | 0.091 | 0.77 | | 0.15 | 0.77 | |
| Thoughts/emotions/actions | 0.009 | 0.99 | -0.085 | 0.77 | 0.081 | 0.77 | | 0.18 | 0.77 | |
| Spatial coherence index | 0.023 | 0.99 | 0.13 | 0.77 | -0.12 | 0.77 | | -0.22 | 0.77 | |
|  |  |  |  |  |  |  | |  |  | |
| **Autobiographical interview** | | | | | | | | | | |
| Internal details | -0.16 | 0.75 | -0.15 | 0.75 | -0.13 | 0.75 | | 0.010 | 0.99 | |
| Internal events | -0.081 | 0.81 | -0.072 | 0.81 | -0.069 | 0.81 | | -0.001 | 1.00 | |
| Internal time | -0.10 | 0.81 | -0.014 | 0.99 | -0.15 | 0.75 | | -0.15 | 0.75 | |
| Internal place | -0.038 | 0.94 | -0.065 | 0.82 | -0.002 | 1.00 | | 0.070 | 0.82 | |
| Internal perceptual | -0.069 | 0.81 | -0.031 | 0.94 | -0.094 | 0.81 | | -0.084 | 0.81 | |
| Internal thoughts/emotions | -0.10 | 0.81 | -0.15 | 0.75 | -0.044 | 0.93 | | 0.11 | 0.81 | |
| Vividness ratings | -0.14 | 0.75 | 0.029 | 0.94 | -0.26 | 0.55 | | -0.28 | 0.55 | |
|  |  |  |  |  |  |  | |  |  | |
| **Future thinking** | | | | | | | | | | |
| Experiential index | 0.18 | 0.39 | 0.16 | 0.49 | 0.16 | 0.49 | | -0.099 | 0.79 | |
| Spatial references | 0.091 | 0.80 | 0.021 | 0.91 | 0.16 | 0.54 | | 0.065 | 0.84 | |
| Entities present | 0.077 | 0.81 | 0.044 | 0.88 | 0.10 | 0.79 | | 0.038 | 0.88 | |
| Sensory descriptions | 0.20 | 0.39 | 0.19 | 0.39 | 0.17 | 0.49 | | -0.087 | 0.80 | |
| Thoughts/emotions/actions | 0.007 | 0.98 | 0.027 | 0.88 | -0.010 | 0.98 | | -0.033 | 0.88 | |
| Spatial coherence index | 0.25 | 0.29 | 0.28 | 0.17 | 0.13 | 0.66 | | -0.22 | 0.35 | |
|  |  |  |  |  |  |  | |  |  | |
| **Navigation** | | | | | | | | | | |
| Overall navigation score | -0.085 | 0.99 | -0.092 | 0.99 | -0.063 | 0.99 | | 0.060 | 0.99 | |
| Scene recognition | -0.023 | 1.00 | -0.035 | 1.00 | -0.008 | 1.00 | | 0.024 | 1.00 | |
| Proximity judgements | -0.097 | 0.99 | 0.003 | 1.00 | -0.21 | 0.99 | | -0.15 | 0.99 | |
| Route knowledge | -0.11 | 0.99 | -0.15 | 0.99 | -0.046 | 0.99 | | 0.134 | 0.99 | |
| Sketch map | -0.084 | 0.99 | -0.068 | 0.99 | -0.084 | 0.99 | | 0.00 | 1.00 | |
|  |  |  |  |  |  |  | |  |  | |

P values are Benjamini-Hochberg false discovery rate corrected at p < 0.05.

**Table S31.** Details of the groups created for each task when taking only the best and worst performers.

| Performance variable | Worst performers maximum | Best performers minimum | N (worst performers) | N (best performers) |
| --- | --- | --- | --- | --- |
|  |  |  |  |  |
| **Scene construction** |  |  |  |  |
| Experiential index | 32.66 | 48.49 | 20 | 20 |
| Spatial references | 1.57 | 5.57 | 21 | 22 |
| Entities present | 6.29 | 13.86 | 20 | 20 |
| Sensory descriptions | 7.71 | 17.0 | 21 | 21 |
| Thoughts/emotions/actions | 1.29 | 6.14 | 19 | 18 |
| Spatial coherence index | 0.71 | 5.0 | 23 | 25 |
|  |  |  |  |  |
| **Autobiographical interview** | | | | |
| Internal details | 15.20 | 34.60 | 22 | 21 |
| Internal events | 6.20 | 16.60 | 21 | 21 |
| Internal time | 2.20 | 10.0 | 18 | 15 |
| Internal place | 1.40 | 3.0 | 26 | 32 |
| Internal perceptual | 0.40 | 2.60 | 20 | 21 |
| Internal thoughts/emotions | 1.20 | 6.0 | 19 | 18 |
| Vividness rating | 3.40 | 5.60 | 19 | 20 |
|  |  |  |  |  |
| **Future thinking** |  |  |  |  |
| Experiential index | 30.13 | 47.33 | 21 | 21 |
| Spatial references | 0.67 | 4.33 | 32 | 30 |
| Entities present | 6.67 | 14.67 | 23 | 22 |
| Sensory descriptions | 4.33 | 13.33 | 21 | 21 |
| Thoughts/emotions/actions | 2.33 | 8.33 | 22 | 22 |
| Spatial coherence index | 0.0 | 5.0 | 23 | 29 |
|  |  |  |  |  |
| **Navigation** |  |  |  |  |
| Overall navigation score | 95.0 | 191.0 | 21 | 21 |
| Scene recognition | 27.0 | 32.0 | 34 | 28 |
| Proximity judgements | 5.0 | 10.0 | 18 | 13 |
| Route knowledge | 6.0 | 19.0 | 23 | 22 |
| Sketch map | 39.0 | 121.0 | 21 | 21 |
|  |  |  |  |  |

**Table S32.** Comparison of hippocampal grey matter MT saturation when taking the best and worst performing participants for task, with age, gender, total intracranial volume and MRI scanner included as covariates.

| Performance variable | Whole hippocampus | | Anterior hippocampus | | Posterior hippocampus | | Posterior/Anterior hippocampus ratio | | |  |
| --- | --- | --- | --- | --- | --- | --- | --- | --- | --- | --- |
|  | F | p | F | p | F | p | | F | p | |
|  |  |  |  |  |  |  | |  |  | |
| **Scene construction** | | | | | | | | | | |
| Experiential index | 2.95 | 0.62 | 2.96 | 0.62 | 1.86 | 0.74 | | 0.18 | 0.85 | |
| Spatial references | 0.76 | 0.74 | 0.57 | 0.75 | 0.69 | 0.74 | | 0.00 | 0.98 | |
| Entities present | 0.26 | 0.82 | 0.68 | 0.74 | 0.00 | 0.98 | | 0.96 | 0.74 | |
| Sensory descriptions | 8.64 | 0.35 | 8.03 | 0.35 | 7.20 | 0.35 | | 1.38 | 0.74 | |
| Thoughts/emotions/actions | 0.91 | 0.74 | 1.47 | 0.74 | 0.37 | 0.78 | | 0.96 | 0.74 | |
| Spatial coherence index | 0.69 | 0.74 | 0.32 | 0.79 | 1.07 | 0.74 | | 0.00 | 0.98 | |
|  |  |  |  |  |  |  | |  |  | |
| **Autobiographical interview** | | | | | | | | | | |
| Internal details | 0.02 | 0.99 | 0.08 | 0.97 | 0.00 | 0.99 | | 0.17 | 0.97 | |
| Internal events | 0.17 | 0.97 | 0.01 | 0.99 | 0.42 | 0.92 | | 0.56 | 0.92 | |
| Internal time | 1.00 | 0.92 | 2.24 | 0.92 | 0.22 | 0.97 | | 1.40 | 0.92 | |
| Internal place | 0.27 | 0.93 | 0.47 | 0.92 | 0.09 | 0.97 | | 0.64 | 0.92 | |
| Internal perceptual | 0.00 | 0.99 | 0.00 | 0.99 | 0.01 | 0.99 | | 0.03 | 0.99 | |
| Internal thoughts/emotions | 0.00 | 0.99 | 0.02 | 0.99 | 0.00 | 0.99 | | 0.04 | 0.99 | |
| Vividness ratings | 2.34 | 0.92 | 2.03 | 0.92 | 2.04 | 0.92 | | 0.03 | 0.99 | |
|  |  |  |  |  |  |  | |  |  | |
| **Future thinking** | | | | | | | | | | |
| Experiential index | 3.77 | 0.48 | 5.01 | 0.30 | 2.30 | 0.59 | | 0.88 | 0.76 | |
| Spatial references | 1.11 | 0.73 | 1.09 | 0.73 | 0.87 | 0.76 | | 0.08 | 0.90 | |
| Entities present | 0.24 | 0.84 | 0.09 | 0.90 | 0.43 | 0.82 | | 0.03 | 0.95 | |
| Sensory descriptions | 0.43 | 0.82 | 0.23 | 0.84 | 0.65 | 0.82 | | 0.14 | 0.88 | |
| Thoughts/emotions/actions | 0.62 | 0.82 | 1.12 | 0.73 | 0.24 | 0.84 | | 0.77 | 0.80 | |
| Spatial coherence index | 0.02 | 0.95 | 0.00 | 0.99 | 0.08 | 0.90 | | 0.02 | 0.96 | |
|  |  |  |  |  |  |  | |  |  | |
| **Navigation** | | | | | | | | | | |
| Overall navigation score | 2.69 | 0.50 | 0.63 | 0.76 | 4.84 | 0.48 | | 3.07 | 0.50 | |
| Scene recognition | 3.35 | 0.48 | 6.75 | 0.48 | 1.19 | 0.70 | | 5.20 | 0.48 | |
| Proximity judgements | 1.58 | 0.68 | 1.04 | 0.73 | 1.89 | 0.62 | | 0.44 | 0.80 | |
| Route knowledge | 0.33 | 0.86 | 0.03 | 0.92 | 0.76 | 0.76 | | 0.87 | 0.73 | |
| Sketch map | 3.82 | 0.48 | 2.87 | 0.50 | 3.42 | 0.48 | | 0.07 | 0.91 | |
|  |  |  |  |  |  |  | |  |  | |

P values are Benjamini-Hochberg false discovery rate corrected at p < 0.05.

**Table S33.** Comparison of hippocampal grey matter PD when taking the best and worst performing participants for task, with age, gender, total intracranial volume and MRI scanner included as covariates.

| Performance variable | Whole hippocampus | | Anterior hippocampus | | Posterior hippocampus | | Posterior/Anterior hippocampus ratio | | |  |
| --- | --- | --- | --- | --- | --- | --- | --- | --- | --- | --- |
|  | F | p | F | p | F | p | | F | p | |
|  |  |  |  |  |  |  | |  |  | |
| **Scene construction** | | | | | | | | | | |
| Experiential index | 0.02 | 0.96 | 0.01 | 0.98 | 0.15 | 0.86 | | 0.35 | 0.79 | |
| Spatial references | 0.13 | 0.88 | 1.55 | 0.74 | 0.37 | 0.78 | | 2.71 | 0.62 | |
| Entities present | 2.69 | 0.62 | 3.42 | 0.62 | 1.04 | 0.74 | | 1.86 | 0.74 | |
| Sensory descriptions | 0.48 | 0.77 | 0.76 | 0.74 | 0.08 | 0.88 | | 0.66 | 0.74 | |
| Thoughts/emotions/actions | 0.45 | 0.77 | 0.09 | 0.88 | 1.17 | 0.74 | | 0.44 | 0.77 | |
| Spatial coherence index | 0.04 | 0.94 | 0.47 | 0.77 | 0.11 | 0.88 | | 1.49 | 0.74 | |
|  |  |  |  |  |  |  | |  |  | |
| **Autobiographical interview** | | | | | | | | | | |
| Internal details | 0.06 | 0.98 | 0.48 | 0.92 | 0.21 | 0.97 | | 1.51 | 0.92 | |
| Internal events | 1.30 | 0.92 | 1.74 | 0.92 | 0.45 | 0.92 | | 1.34 | 0.92 | |
| Internal time | 3.76 | 0.92 | 3.44 | 0.92 | 2.43 | 0.92 | | 1.05 | 0.92 | |
| Internal place | 0.15 | 0.97 | 0.09 | 0.97 | 0.20 | 0.97 | | 0.00 | 0.99 | |
| Internal perceptual | 0.54 | 0.92 | 0.73 | 0.92 | 0.19 | 0.97 | | 0.70 | 0.92 | |
| Internal thoughts/emotions | 1.06 | 0.92 | 1.25 | 0.92 | 0.28 | 0.93 | | 0.56 | 0.92 | |
| Vividness ratings | 2.16 | 0.92 | 1.17 | 0.92 | 2.12 | 0.92 | | 0.00 | 0.99 | |
|  |  |  |  |  |  |  | |  |  | |
| **Future thinking** | | | | | | | | | | |
| Experiential index | 0.87 | 0.76 | 0.97 | 0.76 | 0.42 | 0.82 | | 0.50 | 0.82 | |
| Spatial references | 2.45 | 0.59 | 1.95 | 0.59 | 2.07 | 0.59 | | 0.37 | 0.82 | |
| Entities present | 0.38 | 0.82 | 0.31 | 0.84 | 0.27 | 0.84 | | 0.03 | 0.95 | |
| Sensory descriptions | 0.38 | 0.82 | 0.40 | 0.82 | 0.20 | 0.84 | | 0.29 | 0.84 | |
| Thoughts/emotions/actions | 0.07 | 0.90 | 0.15 | 0.88 | 0.00 | 0.98 | | 0.22 | 0.84 | |
| Spatial coherence index | 0.16 | 0.87 | 0.42 | 0.82 | 0.00 | 0.98 | | 0.41 | 0.82 | |
|  |  |  |  |  |  |  | |  |  | |
| **Navigation** | | | | | | | | | | |
| Overall navigation score | 0.87 | 0.73 | 1.48 | 0.68 | 0.07 | 0.91 | | 1.82 | 0.62 | |
| Scene recognition | 0.02 | 0.92 | 0.06 | 0.91 | 0.00 | 1.00 | | 0.08 | 0.91 | |
| Proximity judgements | 1.34 | 0.68 | 0.49 | 0.80 | 1.95 | 0.62 | | 0.08 | 0.91 | |
| Route knowledge | 0.72 | 0.76 | 1.72 | 0.63 | 0.00 | 0.99 | | 2.63 | 0.50 | |
| Sketch map | 0.20 | 0.89 | 0.00 | 0.99 | 0.87 | 0.73 | | 0.60 | 0.76 | |
|  |  |  |  |  |  |  | |  |  | |

P values are Benjamini-Hochberg false discovery rate corrected at p < 0.05.

**Table S34.** Comparison of hippocampal grey matter R_1_ when taking the best and worst performing participants for task, with age, gender, total intracranial volume and MRI scanner included as covariates.

| Performance variable | Whole hippocampus | | Anterior hippocampus | | Posterior hippocampus | | Posterior/Anterior hippocampus ratio | | |  |
| --- | --- | --- | --- | --- | --- | --- | --- | --- | --- | --- |
|  | F | p | F | p | F | p | | F | p | |
|  |  |  |  |  |  |  | |  |  | |
| **Scene construction** | | | | | | | | | | |
| Experiential index | 1.43 | 0.74 | 2.25 | 0.74 | 0.74 | 0.74 | | 1.60 | 0.74 | |
| Spatial references | 2.89 | 0.62 | 3.62 | 0.62 | 1.91 | 0.74 | | 0.23 | 0.82 | |
| Entities present | 0.08 | 0.88 | 0.00 | 0.98 | 0.23 | 0.82 | | 0.88 | 0.74 | |
| Sensory descriptions | 0.76 | 0.74 | 1.53 | 0.74 | 0.33 | 0.79 | | 0.64 | 0.74 | |
| Thoughts/emotions/actions | 0.41 | 0.78 | 0.93 | 0.74 | 0.12 | 0.88 | | 3.63 | 0.62 | |
| Spatial coherence index | 1.23 | 0.74 | 1.09 | 0.74 | 1.21 | 0.74 | | 0.00 | 0.98 | |
|  |  |  |  |  |  |  | |  |  | |
| **Autobiographical interview** | | | | | | | | | | |
| Internal details | 2.41 | 0.92 | 1.66 | 0.92 | 2.40 | 0.92 | | 0.12 | 0.97 | |
| Internal events | 3.22 | 0.92 | 1.70 | 0.92 | 4.21 | 0.92 | | 0.84 | 0.92 | |
| Internal time | 0.05 | 0.99 | 0.33 | 0.93 | 0.00 | 0.99 | | 0.43 | 0.92 | |
| Internal place | 0.58 | 0.92 | 0.81 | 0.92 | 0.38 | 0.92 | | 0.40 | 0.92 | |
| Internal perceptual | 3.99 | 0.92 | 5.06 | 0.92 | 2.74 | 0.92 | | 0.66 | 0.92 | |
| Internal thoughts/emotions | 0.42 | 0.92 | 0.07 | 0.97 | 0.69 | 0.92 | | 0.45 | 0.92 | |
| Vividness ratings | 0.57 | 0.92 | 0.31 | 0.93 | 0.72 | 0.92 | | 0.12 | 0.97 | |
|  |  |  |  |  |  |  | |  |  | |
| **Future thinking** | | | | | | | | | | |
| Experiential index | 2.04 | 0.59 | 2.57 | 0.59 | 1.24 | 0.73 | | 0.22 | 0.84 | |
| Spatial references | 8.47 | 0.25 | 7.62 | 0.25 | 8.00 | 0.25 | | 0.00 | 0.98 | |
| Entities present | 6.57 | 0.30 | 6.04 | 0.30 | 5.08 | 0.30 | | 0.38 | 0.82 | |
| Sensory descriptions | 3.00 | 0.59 | 2.44 | 0.59 | 2.79 | 0.59 | | 0.03 | 0.95 | |
| Thoughts/emotions/actions | 1.09 | 0.73 | 0.23 | 0.84 | 1.82 | 0.59 | | 2.23 | 0.59 | |
| Spatial coherence index | 0.71 | 0.82 | 0.66 | 0.82 | 0.63 | 0.82 | | 0.01 | 0.98 | |
|  |  |  |  |  |  |  | |  |  | |
| **Navigation** | | | | | | | | | | |
| Overall navigation score | 0.03 | 0.92 | 0.04 | 0.92 | 0.18 | 0.89 | | 0.91 | 0.73 | |
| Scene recognition | 0.06 | 0.91 | 0.25 | 0.88 | 0.00 | 0.99 | | 0.65 | 0.76 | |
| Proximity judgements | 1.35 | 0.68 | 0.89 | 0.73 | 1.31 | 0.68 | | 0.07 | 0.91 | |
| Route knowledge | 0.43 | 0.80 | 1.06 | 0.73 | 0.10 | 0.91 | | 1.34 | 0.68 | |
| Sketch map | 3.97 | 0.48 | 3.54 | 0.48 | 3.62 | 0.48 | | 0.06 | 0.91 | |
|  |  |  |  |  |  |  | |  |  | |

P values are Benjamini-Hochberg false discovery rate corrected at p < 0.05.

**Table S35.** Comparison of hippocampal grey matter R_2_* when taking the best and worst performing participants for task, with age, gender, total intracranial volume and MRI scanner included as covariates.

| Performance variable | Whole hippocampus | | Anterior hippocampus | | Posterior hippocampus | | Posterior/Anterior hippocampus ratio | | |  |
| --- | --- | --- | --- | --- | --- | --- | --- | --- | --- | --- |
|  | F | p | F | p | F | p | | F | p | |
|  |  |  |  |  |  |  | |  |  | |
| **Scene construction** | | | | | | | | | | |
| Experiential index | 5.19 | 0.56 | 4.04 | 0.62 | 4.24 | 0.62 | | 0.09 | 0.88 | |
| Spatial references | 3.02 | 0.62 | 0.46 | 0.77 | 5.61 | 0.56 | | 1.98 | 0.74 | |
| Entities present | 1.03 | 0.74 | 0.23 | 0.82 | 1.78 | 0.74 | | 0.61 | 0.74 | |
| Sensory descriptions | 0.51 | 0.77 | 0.00 | 0.98 | 1.55 | 0.74 | | 1.87 | 0.74 | |
| Thoughts/emotions/actions | 0.02 | 0.96 | 0.17 | 0.85 | 0.27 | 0.82 | | 0.89 | 0.74 | |
| Spatial coherence index | 0.01 | 0.98 | 0.67 | 0.74 | 1.02 | 0.74 | | 3.23 | 0.62 | |
|  |  |  |  |  |  |  | |  |  | |
| **Autobiographical interview** | | | | | | | | | | |
| Internal details | 0.40 | 0.92 | 0.12 | 0.97 | 0.61 | 0.92 | | 0.15 | 0.97 | |
| Internal events | 0.45 | 0.92 | 0.08 | 0.97 | 0.86 | 0.92 | | 0.63 | 0.92 | |
| Internal time | 2.30 | 0.92 | 2.27 | 0.92 | 1.08 | 0.92 | | 0.29 | 0.93 | |
| Internal place | 0.30 | 0.93 | 0.75 | 0.92 | 0.07 | 0.97 | | 0.60 | 0.92 | |
| Internal perceptual | 2.91 | 0.92 | 4.19 | 0.92 | 0.96 | 0.92 | | 2.09 | 0.92 | |
| Internal thoughts/emotions | 0.03 | 0.99 | 0.07 | 0.97 | 0.37 | 0.92 | | 0.75 | 0.92 | |
| Vividness ratings | 0.95 | 0.92 | 0.37 | 0.92 | 1.01 | 0.92 | | 0.08 | 0.97 | |
|  |  |  |  |  |  |  | |  |  | |
| **Future thinking** | | | | | | | | | | |
| Experiential index | 5.16 | 0.30 | 2.96 | 0.59 | 5.15 | 0.30 | | 0.20 | 0.84 | |
| Spatial references | 2.02 | 0.59 | 2.16 | 0.59 | 1.24 | 0.73 | | 0.13 | 0.88 | |
| Entities present | 4.31 | 0.39 | 2.53 | 0.59 | 5.71 | 0.30 | | 0.09 | 0.90 | |
| Sensory descriptions | 1.91 | 0.59 | 1.42 | 0.71 | 1.76 | 0.60 | | 0.05 | 0.94 | |
| Thoughts/emotions/actions | 1.82 | 0.59 | 0.90 | 0.76 | 2.43 | 0.59 | | 0.53 | 0.82 | |
| Spatial coherence index | 0.47 | 0.82 | 1.38 | 0.71 | 0.00 | 1.00 | | 1.16 | 0.73 | |
|  |  |  |  |  |  |  | |  |  | |
| **Navigation** | | | | | | | | | | |
| Overall navigation score | 0.24 | 0.88 | 0.20 | 0.89 | 1.98 | 0.62 | | 4.93 | 0.48 | |
| Scene recognition | 2.19 | 0.58 | 0.59 | 0.76 | 3.07 | 0.50 | | 0.60 | 0.76 | |
| Proximity judgements | 0.03 | 0.92 | 0.11 | 0.91 | 0.81 | 0.75 | | 0.31 | 0.86 | |
| Route knowledge | 0.42 | 0.80 | 0.16 | 0.90 | 2.97 | 0.50 | | 3.75 | 0.48 | |
| Sketch map | 0.42 | 0.80 | 2.23 | 0.58 | 0.25 | 0.88 | | 4.54 | 0.48 | |
|  |  |  |  |  |  |  | |  |  | |

P values are Benjamini-Hochberg false discovery rate corrected at p < 0.05.
